# Serine synthesis pathway inhibition cooperates with dietary serine and glycine limitation for cancer therapy

**DOI:** 10.1038/s41467-020-20223-y

**Published:** 2021-01-14

**Authors:** Mylène Tajan, Marc Hennequart, Eric C. Cheung, Fabio Zani, Andreas K. Hock, Nathalie Legrave, Oliver D. K. Maddocks, Rachel A. Ridgway, Dimitris Athineos, Alejandro Suárez-Bonnet, Robert L. Ludwig, Laura Novellasdemunt, Nikolaos Angelis, Vivian S. W. Li, Georgios Vlachogiannis, Nicola Valeri, Nello Mainolfi, Vipin Suri, Adam Friedman, Mark Manfredi, Karen Blyth, Owen J. Sansom, Karen H. Vousden

**Affiliations:** 1 The Francis Crick Institute, 1 Midland Road, London, NW1 1AT UK; 2 Cancer Research UK Beatson Institute, Switchback Road, Glasgow, G61 1BD UK; 3 Mechanistic Biology and Profiling, Discovery Sciences, R&D, AstraZeneca, Cambridge, UK; 4 Institute of Cancer Sciences, University of Glasgow, Garscube Estate, Glasgow, G61 1QH UK; 5 The Royal Veterinary College, Hawkshead Lane, Harfield, Herts, AL9 7TA UK; 6 Gastrointestinal Cancer Biology and Genomics Team, Centre for Evolution and Cancer, The Institute of Cancer Research, London, UK; 7 Division of Cancer, Department of Surgery and Cancer, Imperial College London, London, UK; 8 Department of Medicine, The Royal Marsden NHS Foundation Trust, London, UK; 9 Raze Therapeutics, Inc., Cambridge, MA USA

**Keywords:** Cancer, Cancer metabolism, Cancer therapy, Oncology

## Abstract

Many tumour cells show dependence on exogenous serine and dietary serine and glycine starvation can inhibit the growth of these cancers and extend survival in mice. However, numerous mechanisms promote resistance to this therapeutic approach, including enhanced expression of the de novo serine synthesis pathway (SSP) enzymes or activation of oncogenes that drive enhanced serine synthesis. Here we show that inhibition of PHGDH, the first step in the SSP, cooperates with serine and glycine depletion to inhibit one-carbon metabolism and cancer growth. In vitro, inhibition of PHGDH combined with serine starvation leads to a defect in global protein synthesis, which blocks the activation of an ATF-4 response and more broadly impacts the protective stress response to amino acid depletion. In vivo, the combination of diet and inhibitor shows therapeutic efficacy against tumours that are resistant to diet or drug alone, with evidence of reduced one-carbon availability. However, the defect in ATF4-response seen in vitro following complete depletion of available serine is not seen in mice, where dietary serine and glycine depletion and treatment with the PHGDH inhibitor lower but do not eliminate serine. Our results indicate that inhibition of PHGDH will augment the therapeutic efficacy of a serine depleted diet.

## Abstract (web summary)

Dietary serine and glycine starvation has emerged as a potential therapy for cancer. Here, the authors show that inhibition of PHGDH, which mediates the first step in the serine synthesis pathway, improves the therapeutic efficacy of serine depletion diet in mouse xenograft models.

## Introduction

Cancer cells adapt their metabolism to support growth and survival, leading to various dependencies and vulnerabilities that could be targeted for therapy^1^. While these metabolic alterations are dictated by numerous factors, including the genetic alterations in the tumour and the tumour environment or tissue of origin, recent attention has focused on the role of serine metabolism in supporting cancer cell growth^2^. Serine and glycine (which is produced from serine by the SHMT1/2 reaction) contribute to a number of important processes, including protein, nucleotide and lipid synthesis, the generation of antioxidant defense through glutathione and NADPH synthesis and the provision of one-carbon units for the folate cycle and methylation reactions^3^. Consequently, cancer cells have been shown to be highly dependent on serine, and antifolates that include dihydrofolate reductase, thymidylate synthase and glycinamide ribonucleotide formyltransferase inhibitors, have been successfully used as therapeutic drugs^4^. The earliest example, the dihydrofolate reductase inhibitor methotrexate, has been used to treat several different types of cancer including haematological malignancies, breast cancer and osteosarcoma for over 70 years, with newer drugs targeting this pathway under constant development^4–6^. As a non-essential amino acid, serine can be taken up from the extracellular environment or synthesised de novo by cells using the serine synthesis pathway (SSP). Tissue culture experiments have shown that many cancer cells avidly consume serine and depend on an exogenous source of serine for optimal growth^7–10^. However, most cells can adapt to serine starvation by activating flux through the SSP. Serine is an activator of PKM2, the final step in glycolysis, and decreased PKM2 activity under serine depleted conditions allows for the diversion of glycolytic intermediates into the SSP^11,12^. This response is coordinated with an ATF-4 and histone methyltransferase G9A-dependent activation of the three enzymes of the SSP^10,12,13^, which allow most cancer cells to survive and continue to proliferate following serine starvation. The efficacy with which cancer cells can adapt to the loss of exogenous serine depends on several factors. Some cancers acquire an amplification or overexpression of PHGDH – the first step in the SSP – and these cells tend to be less affected by serine starvation^14–16^. Similarly, activation of oncogenes such as *KRAS*, *MYC*, *MDM2* and *NRF2*^10,17–19^ can lead to an increase in SSP enzyme expression, also allowing cells to become resistant to depletion of exogenous serine. Conversely, although the p53 tumour suppressor protein can inhibit PHGDH expression^20^, loss of p53 also makes cells more vulnerable to increased ROS that accompanies the switch to de novo serine synthesis, resulting in a decreased survival in serine free medium^7^.

Several studies have shown that dietary serine limitation can slow tumour growth in vivo^7,17,21–24^. Mice fed with a serine and glycine free diet show a decrease in circulating serine and glycine levels and this approach can retard the development and progression of tumours in both xenograft and genetically engineered mouse models. As predicted by in vitro studies, the survival response to dietary serine and glycine restriction was enhanced by interference with antioxidant defence mechanisms^17^. Activation of KRAS or enhanced expression of PHGDH, on the other hand, resulted in an increase in de novo serine synthesis which limited the response to the serine and glycine free diet^17,23^. Under these conditions, it is possible that inhibition of the SSP will cooperate with dietary serine and glycine restriction to improve therapeutic efficacy. Indeed, a recent study has shown cooperation between deletion of PSAT1 (the second step in the serine synthesis pathway) and dietary serine depletion in the inhibition of Myc-driven liver cancer^25^.

Several small molecule inhibitors of PHGDH that inhibit de novo serine synthesis and retard the growth of cancer cells that are dependent on this pathway have been generated^26–28^. This first generation of inhibitors showed relatively weak potency, leading to the development of more active, second generation inhibitors, which were effective to limit cancer cell growth in vitro under serine deprivation but were not suitable for use in vivo^29^. More recently, a highly potent, selective and reversible PHGDH inhibitor, PH755, has been reported to prevent the flux of labelled glucose into serine in LPS stimulated macrophages and improve the survival of mice exposed to LPS-induced endotoxemia^30^. This inhibitor has also been shown to be effective to limit tumour growth in vivo^24,31^. In this study we examine the cooperation between serine and glycine starvation (-SG) and PHGDH inhibition (PHGDHi), demonstrating a reduction of tumour cell growth in vitro and in vivo. Our data support the therapeutic potential of combining dietary serine and glycine restriction with a small molecule inhibitor of PHGDH.

## Results

### PHGDHi/-SG impedes growth of tumour cell lines

**Fig. 1:**
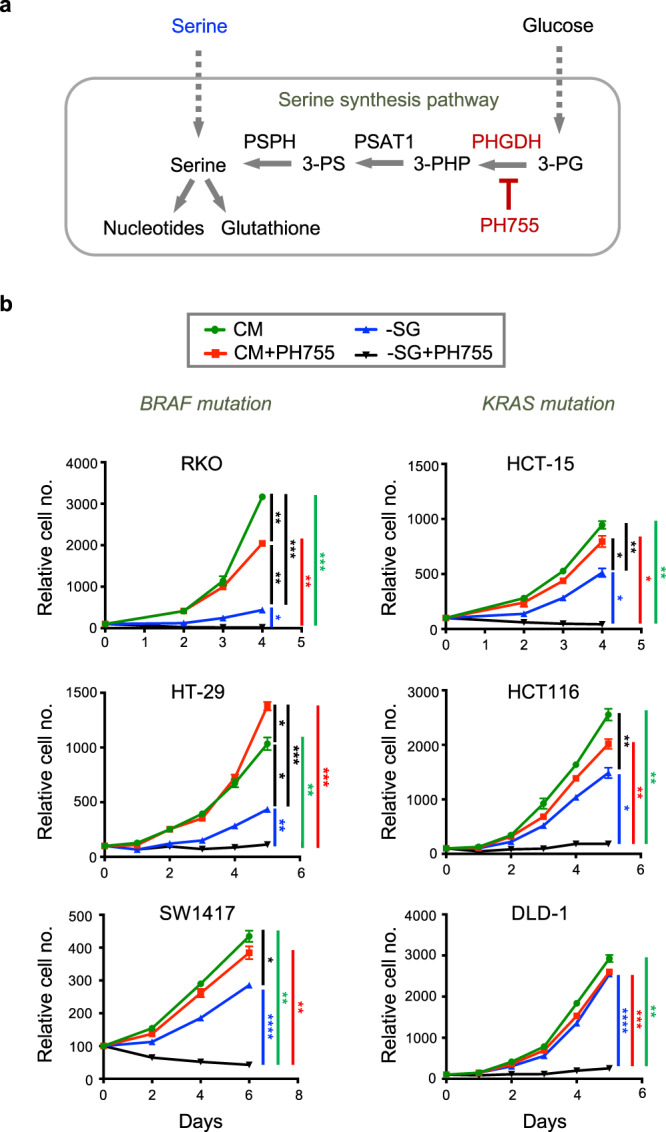
PHGDH inhibition cooperates with serine/glycine depletion to impede tumour growth in vitro. **a** As a non-essential amino acid, serine can be taken up from the environment or newly synthesised through the serine synthesis pathway (SSP). The SSP consists of a three-step enzymatic reaction starting with the NAD^+^-dependent oxidation of the glycolytic intermediate 3-phosphoglycerate (3-PG) to 3-phosphohydroxypyruvate (3-PHP). This first reaction is catalysed by phosphoglycerate dehydrogenase (PHGDH), an enzyme that can be efficiently targeted by the pharmacological compound PH755. The 3-PHP produced during the PHGDH reaction is then converted into 3-phosphoserine (3-PS) by phosphoserine aminotransferase 1 (PSAT1) in a glutamate-dependent transamination reaction. Finally, phosphoserine phosphatase (PSPH) catalyses the hydrolysis of 3-PS to produce serine. Serine is involved in numerous metabolic pathways including nucleotide synthesis or glutathione synthesis, a major antioxidant for the cells. Serine availability can thus be targeted by depleting it from the extracellular environment or by inhibition of the SSP using PH755. **b** Growth curves of colon cancer cell lines grown in complete medium (CM) or equivalent medium lacking serine and glycine (-SG) and treated or not with 10 µM PH755. Data are presented as mean ± SEM of triplicate cultures and are representative of at least two independent experiments (*p < 0.05, **p < 0.01, ***p < 0.001, ****p < 0.0001; two-way ANOVA with Tukey’s post hoc test).

Cells can take up exogenous serine or synthesise serine from the glycolytic intermediate 3-phosphoglycerate (3-PG), using the serine synthesis pathway (Fig. 1a). To assess the relative contribution of each of these pathways to the growth of cells in culture, we measured the proliferation of a series of colorectal cancer cell lines grown in complete medium (CM), medium lacking serine and glycine (-SG), CM with PH755 (a PHGDH inhibitor) or a combination of -SG plus PH755. As noted previously^14,27^, the response to serine and glycine starvation varied between cell lines ranging from RKO, HT-29 and SW48 cells that showed a significant dependence on exogenous serine and glycine for proliferation, to DLD-1, LoVo, CACO-2 and MDA-MB-468 cells (a breast cancer line previously shown to carry PHGDH amplification) that were not affected by lack of serine and glycine in the medium (Fig. 1b and Supplementary Figure 1a). There was a trend for colorectal cancer cell lines carrying KRAS mutation (HCT-15, HCT116, DLD-1, LoVo and SW480) to be more resistant to serine and glycine withdrawal compared to those cell lines carrying BRAF mutations (RKO, HT-29, SW1417 and CL-34), although SW620 (KRAS mutant) and VACO5 (BRAF mutant) were exceptions to this trend (Fig. 1b and Supplementary Figure 1a). While these results are broadly in line with the previously reported role of activated KRAS in supporting de novo serine synthesis^17^, it is clear that other genetic alterations – such as amplification of PHGDH in MDA-MB-468 cells^14^ – also contribute to the dependence of cancer cells on a supply of exogenous serine. Treatment of the cells with PH755 in complete medium had no clear effect on the proliferation rate of the cells, indicating that at this concentration, the inhibitor has no non-specific inhibitory effect on cell growth. However, combining -SG medium with PH755 completely inhibited the growth of all the cell lines tested (Fig. 1b and Supplementary Figure 1a).

### PHGDHi/-SG limits DNA synthesis, survival and organoid growth

**Fig. 2:**
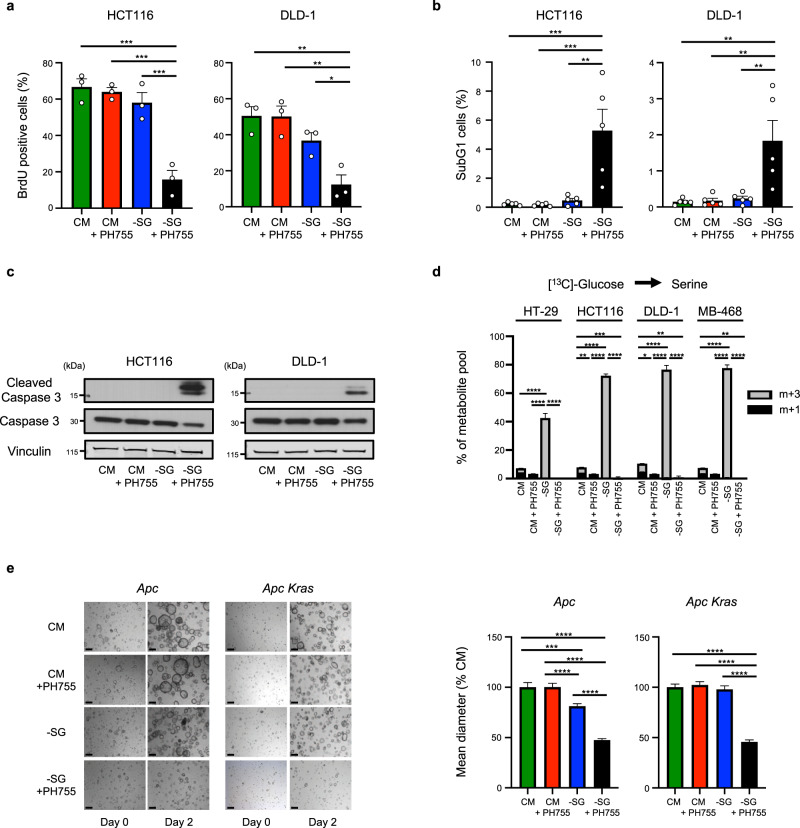
PHGDH inhibition cooperates with serine/glycine depletion to limit DNA synthesis, survival and organoid growth. **a** Percentage of BrdU positive cells in HCT116 and DLD-1 cells grown in CM or -SG medium ±10 µM PH755 for 48 h followed by a 5-h incubation with 10 µM BrdU. Data represents mean ± SEM of three independent experiments (*p < 0.05, **p < 0.01, ***p < 0.001, one-way ANOVA with Tukey’s post hoc test). **b** Percentage of SubG1 cells in HCT116 and DLD-1 cells grown in CM or -SG medium ±10 µM PH755 for 48 h. Data represents mean ± SEM of five independent experiments (**p < 0.01, ***p < 0.001, one-way ANOVA with Tukey’s post hoc test). **c** Cells were grown in CM or -SG medium supplemented or not with 10 µM PH755 for 2 days (HCT116) or 3 days (DLD-1). Western blots show the expression of cleaved Caspase-3 and Caspase-3. Membrane was reprobed with vinculin as a loading control. Data are representative of three independent experiments. **d** Intracellular serine level in cells grown in CM or -SG medium ±10 µM PH755 containing U-[^13^C]-glucose was measured by LC-MS. Metabolite percentages are represented as mean ± SEM of triplicate cultures and are representative of three independent experiments (*p < 0.05, **p < 0.01, ***p < 0.001, ****p < 0.0001; one-way ANOVA with Tukey’s post hoc test). **e** Intestinal tumour organoids derived from *Vil1-creER;Apc*^*fl/fl*^ (*Apc*) and *Vil1-creER;Apc*^*fl/fl*^*;Kras*^*G12D/+*^ (*Apc Kras*) mice were grown in CM or -SG medium supplemented or not with 10 µM PH755. Left panel: representative pictures of the organoids are shown before (day 0) and 2 days after medium change. Right panel: quantification of organoid diameter 2 (*Apc*) or 4 days (*Apc Kras*) after medium change. Data are presented as mean ± SEM (*n* = number of organoids measured per condition; *Apc*: CM: *n* = 113, CM + PH755: *n* = 200, -SG: *n* = 190, -SG + PH755: *n* = 158; *Apc Kras:* CM: *n* = 149, CM + PH755: *n* = 134, -SG: *n* = 134, -SG + PH755: *n* = 78) and are representative of at least two independent experiments (***p < 0.001, ****p < 0.0001; one-way ANOVA with Tukey’s post hoc test). Scale bar represents 200 μm.

Accompanying the lack of proliferation was a strong reduction of BrdU incorporation into newly synthesised DNA after 48 h incubation with -SG medium plus PH755, compared to either treatment alone (Fig. 2a and Supplementary Figure 1b). The decrease in cells undergoing S-phase was accompanied by an accumulation of cells in G2/M phase (Supplementary Figure 1b and Supplementary Figure 1c) and an increase in the proportion of SubG1 cells in the double-treated condition, indicating an increase in cell death (Fig. 2b). The appearance of cleaved caspase-3 confirmed the induction of apoptosis in cells cultured in -SG medium and treated with PH755 (Fig. 2c). Using uniformly labelled glucose, we showed that cells grown in the presence of exogenous serine diverted little glucose into serine and glycine synthesis, as reflected by the negligible accumulation of m + 3 serine and m + 2 glycine (Fig. 2d and Supplementary Figure 1d), regardless of the presence or absence of PH755. Of note, these cells maintained much higher overall intracellular serine and glycine levels than cells grown in the -SG medium (Supplementary Figure 1e). When starved of serine and glycine, all the cell lines showed a clear increase in de novo serine synthesis, as indicated by the accumulation of m + 3 labelled serine and m + 2 labelled glycine (Fig. 2d and Supplementary Figure 1d). Interestingly, this response was weaker in the HT-29 cells, consistent with their lower ability to proliferate in the absence of exogenous serine. However, treatment of the cells with PH755 completely blocked de novo synthesis of serine and glycine, both in complete medium and under serine and glycine starvation (Fig. 2d and Supplementary Figure 1d), demonstrating the efficiency of this inhibitor in blocking PHGDH activity and the SSP. To further verify the specificity of PH755, we tested the effect of genetic deletion of *PHGDH*. DLD-1 cells showed a strong induction of PHGDH expression in response to serine and glycine starvation, which was much less robust in HT-29 cells – consistent with the relative ability of these cell lines to proliferate in the absence of exogenous serine and glycine (Supplementary Figure 1f). Proliferation of these cells following CRISPR-mediated deletion of PHGDH (Supplementary Figure 1f) essentially mirrored that seen following PH755 treatment (Fig. 1b), supporting the function of PH755 as an inhibitor of PHGDH.

Cells grown in 2D on plastic can show different metabolic requirements compared to cells grown under more physiologically relevant conditions, and so we examined the effect of serine and glycine depletion and PH755 treatment on intestinal tumour organoids derived from *Vil1-creER;Apc*^*fl/fl*^ (*Apc*) or *Vil1-creER;Apc*^*fl/fl*^*;Kras*^*G12D/+*^ (*Apc Kras*) mice (Fig. 2e and Supplementary Figure 2a). As shown previously, organoids derived from *Apc* mutant tumours showed some sensitivity to serine and glycine depletion, which was not evident in *Apc/Kras* mutant organoids. Consistent with the observations in 2D cell lines, treatment with PH755 alone did not impact the growth of *Apc* or *Apc/Kras* organoids. However, the combination of serine and glycine starvation and PH755 treatment very effectively inhibited the growth of both *Apc* and *Apc/Kras* organoids (Fig. 2e and Supplementary Figure 2a). Of note, this effect was not restricted to cancer-derived intestinal organoids, as a substantial reduction in growth was also observed in normal small intestine organoids treated with the combination treatment (Supplementary Figure 2b). To validate the effect of the double treatment in human cells, we tested four patient-derived colorectal cancer organoids with different KRAS status (C-001: WT, C-004: deletion, R-006: Gly12Asp and R-008: Gly13Asp)^32^. While -SG or PH755 treatment alone did not have any obvious impact on proliferation, in each case, the combination of drug and inhibitor greatly decreased organoid growth, regardless of KRAS status (Supplementary Figure 2c). While we see a trend for cells with KRAS mutations to be less sensitive to serine and glycine starvation alone, exceptions are likely to reflect other changes that influence the serine synthesis pathway such as PHGDH amplification.

### PHGDHi/-SG treatment inhibits purine and GSH synthesis

**Fig. 3:**
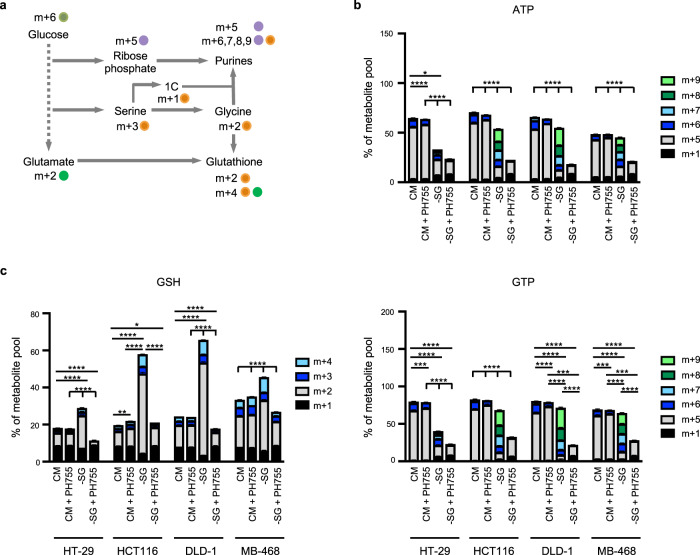
Combining PHGDH inhibition with serine/glycine depletion inhibits purine and glutathione synthesis. **a** Scheme representing the fate of uniformly carbon labelled glucose (m + 6) into purine and glutathione synthesis. Glucose is converted through the pentose phosphate pathway into ribose-5-phosphate, a five-carbon sugar (m + 5), that will be added to purine bases to form purine nucleosides. Purine rings also contain two one-carbon units and an intact glycine that can both come from serine metabolism. Serine is synthesised from the glycolytic intermediate 3-PG, producing an m + 3 isotopomer from uniformly labelled glucose. Serine (m + 3) can generate labelled glycine (m + 2) and labelled one-carbon units (m + 1). The combination of labelled ribose phosphate, glycine and one-carbon units can thus generate m + 5 and greater labelled purines. While m + 5 labelled purines represent a contribution of glucose to ribose synthesis alone, m + 6–9 labelled purines are likely to represent a contribution from de novo synthesised serine. Glutathione is made from glycine, glutamate (both can be m + 2 labelled from glucose) and cysteine. The main isotopomer (m + 2) of glutathione is likely to be derived from m + 2 glycine with the m + 4 labelling reflecting incorporation of m + 2 glycine and m + 2 glutamate. **b**, **c** Intracellular ATP and GTP levels (**b**) or GSH level (**c**) in HT-29, HCT116, DLD-1 and MDA-MB-468 cells grown in CM or -SG medium ±10 µM PH755 containing U-[^13^C]-glucose were measured by LC-MS. Metabolite percentages are represented as mean ± SEM of triplicate cultures and are representative of three independent experiments. Statistics have been performed by comparing the sum of m + 6–9% of metabolite pool for ATP and GTP and the sum of m + 2–4% of metabolite pool for GSH between experimental groups (*p < 0.05, **p < 0.01, ***p < 0.001, ****p < 0.0001; one-way ANOVA with Tukey’s post hoc test).

Serine is involved in numerous metabolic pathways, including the provision of one-carbon units and glycine for purine synthesis and the maintenance of redox homeostasis through glutathione production. The contribution of de novo synthesised serine to these pathways can be assessed by following the fate of uniformly carbon labelled glucose (Fig. 3a). As expected, cells grown in the presence of serine and glycine showed little evidence of the use of de novo synthesised serine for ATP or GTP synthesis (Fig. 3b), the majority of label (m + 5) deriving from ribose synthesised through the pentose phosphate pathway (Fig. 3a). Under serine and glycine starvation, cells that were best able to adapt to these conditions (HCT116, DLD-1 and MDA-MB-468) accumulated m + 6 to m + 9 labelled purines, consistent with the incorporation of labelled serine generated through the SSP (Fig. 3b). As expected, serine and glycine starvation with PH755 treatment effectively inhibited synthesis of ATP and GTP (Fig. 3b). Glutathione can be labelled from glucose derived glycine (m + 2) or glutamate (m + 2; Fig. 3a), although under these conditions the generation of m + 2 glutamate was not impacted by PH755 treatment in most of the cell lines tested (Supplementary Figure 3a). The increase in the proportion of m + 2 and m + 4 labelled glutathione detected in response to the removal of exogenous serine and glycine is therefore likely to reflect the increase in SSP activity and production of labelled glycine, a response that was blocked by treatment with PH755 (Fig. 3c). Importantly, the inability of the double-treated cells to newly synthetise purines and glutathione was evident as early as 3- or 6-h post-treatment, demonstrating that this response represents a primary effect of the combination treatment (Supplementary Figure 3b). Of note, total purine and GSH levels were not decreased in the double-treated cells compared to the cells grown in -SG medium, probably reflecting the lack of consumption of these metabolites when proliferation is inhibited (Supplementary Figure 3c). These results confirm that metabolic pathways that are dependent on serine and critical for the growth of cancer cells are efficiently inhibited by a combination of serine and glycine starvation and the PHGDH inhibitor.

### Metabolic rescue of PHGDHi/-SG treated cells

**Fig. 4:**
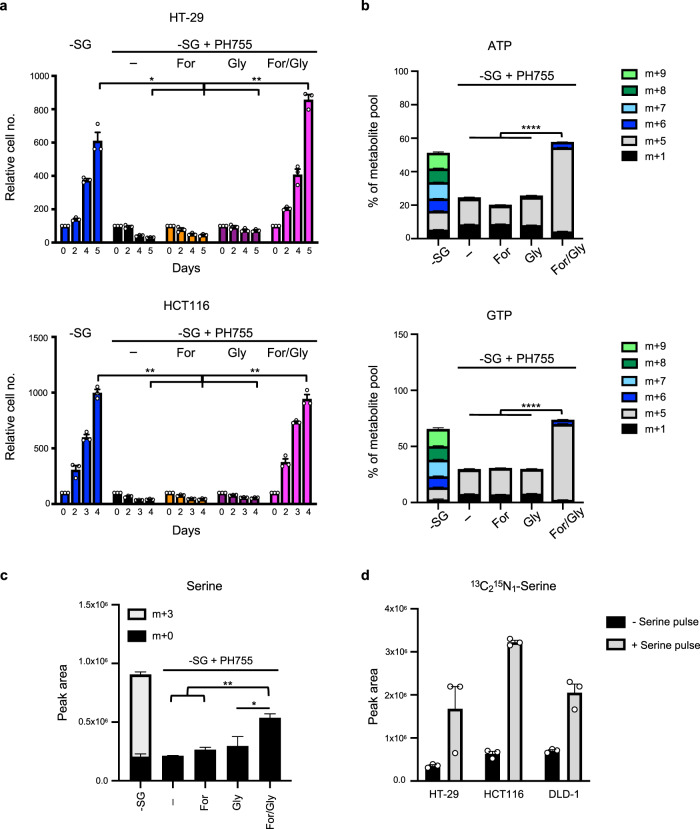
Formate and glycine supplementation rescues cancer cells grown in serine/glycine free medium in presence of the PHGDH inhibitor. **a** Proliferation assay of HT-29 and HCT116 cells grown in -SG medium or -SG medium + 10 µM PH755 supplemented or not with 1 mM sodium formate (For), 0.4 mM glycine (Gly) or both (For/Gly). Data are presented as mean ± SEM of triplicate cultures and are representative of three independent experiments (*p < 0.05, **p < 0.01; two-way ANOVA with Tukey’s post hoc test). **b**, **c** HCT116 cells were grown in -SG medium or -SG medium + 10 µM PH755 supplemented or not with 1 mM sodium formate (For), 0.4 mM glycine (Gly) or both (For/Gly) in presence of U-[^13^C]-glucose. **b** ATP and GTP levels were measured by LC-MS. Metabolite percentages are represented as mean ± SEM of triplicate cultures and are representative of two independent experiments. **c** Serine level was measured by LC-MS. Data are presented as mean ± SEM of triplicate cultures and are representative of two independent experiments (*p < 0.05, **p < 0.01, ****p < 0.0001; one-way ANOVA with Tukey’s post hoc test). **d** HT-29, HCT116 and DLD-1 cells were grown in -SG medium + 10 µM PH755 supplemented with 1 mM sodium formate and 0.4 mM glycine for 24 h in presence of ^13^C_2_^15^N_1_-Glycine for the last hour. ^13^C_2_^15^N_1_-Serine intracellular level was measured by LC-MS after adding a pulse of unlabelled 1 mM serine in the extracellular medium (+serine pulse) or not (-serine pulse) 1 min before metabolite extraction. Data are presented as mean ± SEM of triplicate wells and are representative of three independent experiments.

All cells deprived of serine and glycine and treated with PH755 showed a strong growth inhibition (Figs. 1b, 2e, Supplementary Figure 1a and Supplementary Figure 2a–c). While supplementation of the double-treated cells with either formate (to replenish the one-carbon cycle) or glycine alone did not restore growth, addition of formate and glycine effectively rescued proliferation (Fig. 4a). This proliferation rescue was accompanied by the recovery of ATP and GTP synthesis (Fig. 4b), and the partial restoration of the pool of unlabelled serine (Fig. 4c). Using labelled glycine, we were able to demonstrate that this pool of serine is generated from glycine and one-carbon units provided by formate, a response that is made more evident following the addition of a pulse of unlabelled serine to allow the labelled serine to accumulate (Fig. 4d). These results show that the inhibition of proliferation is a direct effect of inhibition of de novo serine synthesis by PH755, and not a response to any off-target toxicity. The specificity of the metabolic defect induced by PH755 was further supported by the similarity of the response to genetic deletion of *PHGDH* (Supplementary Figure 4a–c).

### PHGDHi/-SG treatment impairs the general ATF-4 response

**Fig. 5:**
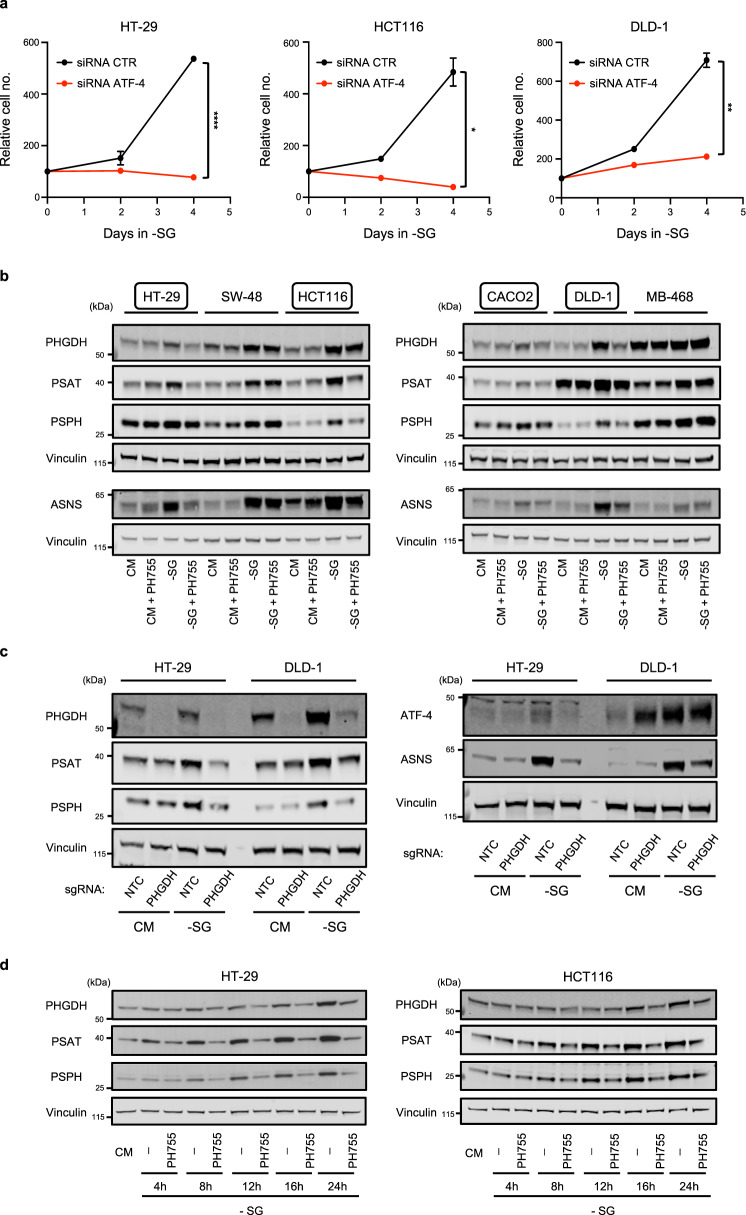
Combining PHGDH inhibition with serine/glycine depletion impairs the general ATF-4 response to amino acid starvation. **a** Growth curves of HT-29, HCT116 and DLD-1 cells transiently depleted of ATF-4 using short interfering RNA (siRNA) and cultured in -SG medium for 4 days. Data are presented as mean ± SEM of triplicate cultures and are representative of two independent experiments (*p < 0.05, **p < 0.01, ****p < 0.0001; two-way ANOVA with Sidak’s post hoc test). **b** Cells were grown in CM or -SG medium supplemented or not with 10 µM PH755 for 24 h. Western blots show the expression of the three SSP enzymes PHGDH, PSAT and PSPH (membrane was reprobed with vinculin as a loading control) or the expression of the ATF-4 target ASNS (membrane was reprobed with vinculin as a loading control). Data are representative of at least two independent experiments. **c** HT-29 and DLD-1 cells infected with Cas9/PHGDH single guide RNA (sgRNA) were cultured in CM or -SG medium for 24 h. Western blots show expression of PHGDH, PSAT and PSPH (membrane was reprobed with vinculin as a loading control) or expression of ATF-4 and ASNS (membrane was reprobed with vinculin as a loading control) in these cells. Data are representative of three independent experiments. **d** HT-29 and HCT116 cells were grown in CM or -SG medium supplemented or not with 10 µM PH755 for 4 h, 8 h, 12 h, 16 h and 24 h. Western blots show SSP enzymes expression in these cells. Each membrane was reprobed with vinculin as a loading control. Data are representative of two independent experiments.

While the effect of PH755 was consistent with a specific inhibition of PHGDH, analysis of the expression of the serine synthesis pathway enzymes in response to PH755 treatment revealed an unexpected response in some of the cell lines. Serine and glycine starvation has been shown to lead to the activation of ATF-4, which can mediate a general survival response to metabolic stress. Importantly, serine starvation leads to an ATF-4 dependent induction of expression of the SSP enzymes, so contributing to the ability of the cells to adapt to a reduction in exogenous serine levels^12^. We confirmed the importance of this response by showing that depletion of ATF-4 resulted in an inability of cells to adapt and grow under serine and glycine starvation (Fig. 5a). As expected, serine and glycine depletion led to an induction of expression of all three SSP enzymes in all the cell lines tested, although this was less robust in MDA-MB-468 cells that constitutively overexpress these enzymes (Fig. 5b). However, in four of the colon cancer lines (HT-29, HCT116, CACO2 and DLD-1), further treatment of serine and glycine starved cells with PH755 diminished this increase in SSP enzyme expression (Fig. 5b), although this was not seen in SW48 cells. A similar response following serine and glycine starvation and PHGDH deletion confirmed that this was a response to loss of PHGDH activity (Fig. 5c). While the decrease in activation of the SSP enzymes is correlated with the growth inhibition seen following serine and glycine starvation and PH755 treatment, the failure of doubly treated cells to induce the SSP enzymes is evident within 4–8 h of serine and glycine starvation (Fig. 5d), suggesting this is a direct response to PHGDH inhibition rather than an indirect response to growth arrest. The loss of ability to induce SSP enzyme expression in response to serine and glycine starvation was accompanied by a general inability to activate an ATF-4 response, as measured by a lack of induction of the canonical ATF-4 target, ASNS (Fig. 5b, c). These results suggest that cells can respond to the combination of serine starvation and SSP inhibition differently than to either intervention alone.

### PHGDHi/-SG treatment inhibits global protein synthesis

**Fig. 6:**
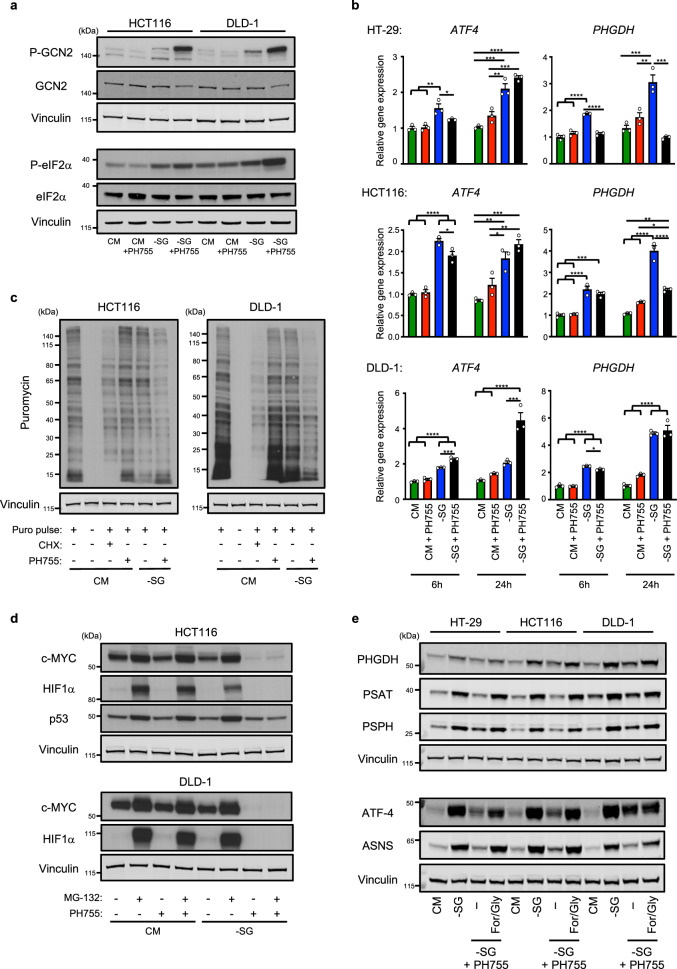
Combining PHGDH inhibition with serine/glycine depletion inhibits global protein synthesis. **a** Cells were grown in CM or -SG medium supplemented or not with 10 µM PH755 for 24 h. Western blots show Phospho-GCN2 (Thr899), GCN2, Phospho-eIF2α (Ser51) and eIF2α. Membranes were reprobed with vinculin as a loading control. Data are representative of two independent experiments. **b** Cells were grown in CM or -SG medium supplemented or not with 10 µM PH755 for 6 h or 24 h. Relative gene expression of *ATF4* and *PHGDH* were measured by qPCR and normalised to the cells grown in CM for 6 h. Data are presented as mean ± SEM of triplicate cultures and are representative of two independent experiments (*p < 0.05, **p < 0.01, ***p < 0.001, ****p < 0.0001; one-way ANOVA with Tukey’s post hoc test). **c** Cells were grown in CM or -SG medium supplemented or not with 10 µM PH755 for 24 h. Puromycin (90 µM) was added in culture medium 10 min before harvesting the cells. When indicated, cells were treated with 10 µg/mL cycloheximide (CHX), a well-known protein synthesis inhibitor, 5 h before harvesting the cells. Western blots show puromycylated peptides. Membrane was reprobed with vinculin as a loading control. Data are representative of two independent experiments. **d** Cells were grown in CM or -SG medium supplemented or not with 10 µM PH755 for 24 h. When indicated, cells were treated with 10 µM MG-132, a proteasome inhibitor, 6 h before harvesting the cells. Western blots show the expression of c-MYC, HIF1α and p53. Membrane was reprobed with vinculin as a loading control. Data are representative of three independent experiments. **e** Cells were grown in CM, -SG medium or -SG medium +10 µM PH755 supplemented or not with 1 mM sodium formate plus 0.4 mM glycine (For/Gly). Western blot shows the expression of the three SSP enzymes PHGDH, PSAT and PSPH or the expression of ATF-4 and its canonical target ASNS after a 24 h incubation in these medium. Membrane was reprobed with vinculin as a loading control. Data are representative of two independent experiments.

To explore how PHGDH activity affects the ATF-4 response induced following serine and glycine withdrawal, we examined the level of activation of the upstream regulators responsible for ATF-4 induction. In response to amino acid starvation, the accumulation of uncharged tRNA leads to the activation and autophosphorylation of the kinase General Control Non-derepressible 2 (GCN2)^33^. GCN2 then phosphorylates the eukaryotic translation initiation factor 2 (eIF2α) at serine 51, leading to a general downregulation of global protein synthesis but selectively inducing the translation of ATF-4^34^. As expected, serine and glycine withdrawal induced the phosphorylation of GCN2 and its target eIF2α in HCT116 and DLD-1 cells (Fig. 6a). Interestingly, this induction of GCN2 and eIF2α phosphorylation was sustained or even more pronounced in cells co-treated with PH755 (Fig. 6a), demonstrating that the lack of ATF-4 upregulation in the double-treated cells was not due to a lack of activation of its upstream regulators. ATF-4 protein levels can also be regulated through ubiquitin-dependent proteasomal degradation^35,36^. However, while treatment of cells with the proteasome inhibitor MG-132 led to a strong accumulation of ATF-4 in cells grown in complete medium, there was no restoration of ATF-4 protein levels or expression of target gene products ASNS and PSAT in cells grown in -SG medium plus PH755 (Supplementary Figure 5a). Furthermore, gene expression analysis showed that *ATF4* was induced at the transcriptional level by 24 h after serine and glycine deprivation regardless of the presence of the PHGDH inhibitor (Fig. 6b). These data indicate that the lack of ATF-4 induction in double-treated cells was not due to increased protein degradation or decreased transcription. As expected, the transcription of ATF-4 target genes encoding ASNS and the SSP enzymes was strongly up-regulated in serine and glycine starved cells (Fig. 6b and Supplementary Figure 5b). Interestingly, the transcription of ATF-4 target genes reflected the extent of ATF-4 induction in the different cell lines grown in -SG medium plus PH755. DLD-1 cells, which maintained some induction of ATF-4 under these conditions (Fig. 6e), retained the ability to induce expression of *PHGDH, ASNS, PSAT* and *PSPH* (Fig. 6b and Supplementary Figure 5b) while HT-29 and HCT116 cells, which showed a more blunted induction of ATF-4 (Fig. 6e) also showed a more severe defect in the ability to transcriptionally activate these ATF-4 target genes (Fig. 6b and Supplementary Figure 5b). We therefore concluded that PH755 treatment did not primarily affect the transcription of ATF-4 or ATF-4 target genes, but impacted the subsequent expression of each of these proteins. To determine whether this reflected a general inhibition of translation resulting from the dramatic decrease of serine and glycine availability seen in this condition, we looked at the incorporation of puromycin, a tyrosyl-tRNA mimetic, into newly synthesised polypeptides^37^ in cells grown in CM or -SG medium plus PH755. Interestingly, while we observed a modest decrease in the amount of puromycin-labelled peptides in response to serine/glycine withdrawal, this reduction was much more pronounced in presence of the PHGDH inhibitor (Fig. 6c). Consistent with a global inhibition of translation in the double-treated cells, the ability of proteasome inhibition to drive the accumulation of short-lived proteins such as c-MYC, HIF1α and p53 was fully blocked in -SG plus PH755-treated cells (Fig. 6d). As reported under other conditions that induce a general inhibition of protein synthesis (such as cycloheximide or puromycin treatment)^38^, mTORC1 is hyper-activated in the double-treated cells, as shown by the accumulation of phosphorylated S6K (Supplementary Figure 5c). Therefore, it seems that the lack of serine and glycine availability triggered by the inhibition of both extracellular and intracellular supplies of these amino acids (Supplementary Figure 1e) interrupts normal translation and prevents the induction of an ATF-4-mediated protective response. In support of this model, we observed that the effect of PHGDH inhibition on the ATF-4 response was specific to serine and glycine deprivation, since treatment with PH755 did not prevent the induction of ATF-4 targets in response to ER stress (Supplementary Figure 5d). Furthermore, supplementation of the double-treated cells with formate and glycine – a treatment that restored some level of serine availability (Fig. 4c) – fully rescued the ATF-4 response (Fig. 6e). Therefore, in the absence of extracellular serine, PHGDH activity becomes essential to maintain global protein synthesis, allowing the induction of a protective ATF-4 response.

### Combining -SG diet and PHGDHi is well-tolerated in vivo

Our in vitro data indicated that the growth inhibitory response to serine and glycine depletion is greatly augmented by treatment of cells with the PHGDH inhibitor, prompting us to test the efficacy of this approach in vivo. Previous reports have shown that our serine- and glycine-free (-SG) diet can lead to a modest drop in body weight in mice^7^ and we showed inhibition of both normal and cancer intestinal organoid growth in response to double treatment in vitro (Supplementary Figure 2). To assess the tolerability of dietary serine/glycine limitation with PH755 treatment, we examined the response to the various treatments in a cohort of tumour-free immunocompetent C57BL/6J mice. As seen previously^7^, mice moved to the -SG diet showed a slight drop in weight that stabilised over the course of the study (Supplementary Figure 6a). Treatment with PH755 alone did not result in any detectable adverse response in these mice, which did not lose body weight compared to control mice (Supplementary Figure 6a). However, mice co-treated with PH755 and the -SG diet showed greater weight loss compared to either treatment alone (Supplementary Figure 6a), despite remaining active and appearing healthy. The weight loss was highly responsive to the dose of PH755, and modulation of the dose (from 75 to 50 mg/kg) was successful in limiting weight loss to <20% over the course of the study (Supplementary Figure 6a). Serine is important in brain development and function and PHGDH deficiency in humans can lead to neurological defects such as microcephaly, psychomotor retardation and seizures^39^. We therefore assessed the impact of -SG diet and PHGDH inhibitor treatment on the brain morphology of a cohort of C57BL/6J mice after 20 days of treatment. Microscopic examination of coronal sections from the brains of the four groups of mice at the level of the pyriform cortex, caudal diencephalon, caudal mesencephalon and rostral cerebellum did not reveal any histopathological lesions in any of the sections examined. Indeed, Haematoxylin & eosin stained sections exhibit normal histological features with no evidence of degeneration, necrosis or inflammation (Supplementary Figure 6b, c). Furthermore, the brain weight remained unchanged in all groups of mice (Supplementary Figure 6b). We also looked for other signs of toxicity of the double treatment in these normal mice. Measurement of plasma AST and ALT activity at end point did not reveal any significant elevation of these markers of liver toxicity in the group of mice treated with -SG diet and PH755 (Supplementary Figure 6d), while plasma urea and creatinine levels remained normal in the double-treated mice, suggesting that there was no kidney damage (Supplementary Figure 6e).

The only clear deleterious effect of the double treatment in mice was weight loss, and our in vitro work using normal mice organoids derived from small intestine revealed that the combination treatment altered their ability to grow (Supplementary Figure 2b). While serine and glycine starvation or PHGDH inhibition alone had no detectable effect on gut morphology, we did note a significant decrease in the length of intestinal villi in animals co-treated with the -SG diet and PH755 (Supplementary Figure 6f), consistent with the greater weight loss seen in these mice. However, these mice showed no clear defect in crypt proliferation – assessed by Ki-67 staining – (Supplementary Figure 6g), suggestive of relatively unperturbed crypt homeostasis. These results are consistent with the observation that reduction in the dose of PHGDH inhibitor stops further weight loss and suggest that short-term combination treatment does not cause long-term damage.

### Combining -SG diet and PHGDHi impedes tumour growth in vivo

**Fig. 7:**
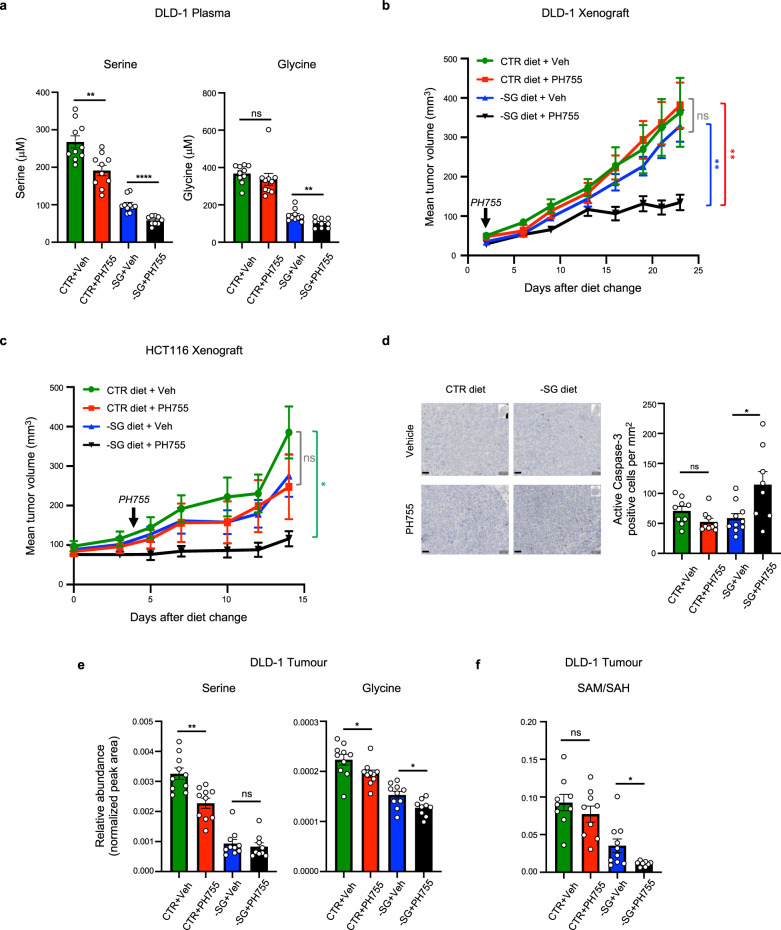
Dietary restriction of serine and glycine and PHGDH inhibition synergizes to deplete circulating and intratumor levels of these amino acids to inhibit tumour growth in xenograft models of colorectal cancer. **a** Serine and glycine levels in plasma from DLD-1 tumour-bearing mice fed a control diet (CTR) or an equivalent diet lacking serine and glycine (-SG) and treated with vehicle (Veh) or PH755. CTR + Veh: *n* = 10; CTR + PH755: *n* = 10; -SG + Veh: *n* = 10; -SG + PH755: n = 9. Data are presented as mean ± SEM. (**p < 0.01, ****p < 0.0001, unpaired two-sided Student’s *t* test). **b** Mice subcutaneously injected with DLD-1 cells were moved to CTR or -SG diet 2 days after tumour cell injection. Two days after diet change, mice were dosed with vehicle or PH755 once daily for 20 days. The starting dosage of PH755 (100 mg/kg for 7 days) was lowered to 50 mg/kg (for 6 days) and increased to 75 mg/kg (for 7 days). Tumour volumes are presented as mean ± SEM. CTR + Veh: *n* = 10; CTR + PH755: *n* = 10; −SG + Veh: *n* = 10; -SG + PH755: *n* = 9. (ns: no significance, **p < 0.01; two-way ANOVA plus Tukey’s post hoc test). **c** Mice subcutaneously injected with HCT116 cells were moved to CTR or -SG diet ten days after tumour cell injection. Four days after diet change, mice were dosed with vehicle or PH755 once daily for 11 days. The starting dosage of PH755 (100 mg/kg for 3 days) was lowered to 50 mg/kg (for 8 days). Tumour volumes are presented as mean ± SEM. CTR + Veh: *n* = 8; CTR + PH755: *n* = 7; −SG + Veh: *n* = 8; −SG + PH755: *n* = 7. (ns: no significance, *p < 0.05; two-way ANOVA plus Tukey’s post hoc test). **d** Representative immunohistochemistry pictures and quantification of active Caspase-3 positive cells in DLD-1 tumours harvested at end-points from mice fed a CTR or -SG diet and treated with vehicle or PH755. CTR + Veh: *n* = 9; CTR + PH755: *n* = 9; -SG + Veh: *n* = 10; -SG + PH755: *n* = 8. Data are presented as mean ± SEM. (*p < 0.05; unpaired two-sided Student’s *t* test with Welch’s correction). Scale bar represents 50 μm. **e**, **f** Serine, glycine, SAM/SAH ratios in tumour lysates. Peak area was normalised to total ion count. **e** CTR + Veh: *n* = 10; CTR + PH755: *n* = 10; -SG + Veh: *n* = 10; -SG + PH755: *n* = 9. **f** CTR + Veh: *n* = 9; CTR + PH755: *n* = 9; -SG + Veh: *n* = 10; -SG + PH755: *n* = 8 (*p < 0.05, **p < 0.01; unpaired two-sided Student’s *t* test with Welch’s correction applied when necessary). (*n* = number of mice).

To explore the antitumor efficacy of the combination therapy, we used xenograft models with two of the colon cancer cell lines that had been tested in vitro, DLD-1 and HCT116. Following subcutaneous injection of cells, mice were transferred to a -SG or control diet when tumours started to become evident and treated with PH755 2–4 days later. As seen in the non-tumour-bearing mice, the double-treated DLD-1 tumour-bearing mice showed more weight loss compared to either treatment alone but a careful modulation of the dose of PH755 used in association with the -SG diet was able to limit the weight loss in these mice to <20% over the course of the experiment (Supplementary Figure 7a). This enhanced weight loss was avoided by increasing the time between diet change and PH755 treatment from 2 to 4 days in the HCT116 experiment (Supplementary Figure 7a, b). Analysis of the circulating amino acid levels at the end point of the studies confirmed previous observations that the -SG diet resulted in a decrease in plasma serine and glycine levels (Fig. 7a and Supplementary Figure 7c). While treatment of mice with PH755 had a more modest effect on circulating serine and glycine, a combination of the -SG diet and PH755 most effectively lowered plasma serine and glycine levels reaching absolute concentration as low as 58 μM serine (versus 267.7 μM in control mice and 99.9 μM in mice fed a -SG diet only) and 102.3 μM glycine (versus 367.6 μM in control mice and 143.6 μM in mice fed a -SG diet only; Fig. 7a and Supplementary Figure 7c). The growth of tumours arising from DLD-1 cells was not affected by dietary intervention or PH755 treatment alone (Fig. 7b), consistent with the lack of effect of either of these treatments on the proliferation of these cells in vitro (Fig. 1b). However, a combination of diet and PH755 strongly inhibited the growth of these tumours (Fig. 7b). The growth of HCT116 xenograft tumours was somewhat sensitive to dietary serine and glycine restriction (as previously shown^7^) and also showed a trend to a decrease in mice treated with PH755 (Fig. 7c), consistent with a previous report showing an effect of PH755 on HCT116 tumour growth^24^. However, the combination treatment of diet and PH755 almost completely blocked the growth of these tumours (Fig. 7c). Interestingly, the strong growth inhibition observed in the double-treated tumours was accompanied by an increased cell death, as reflected by an increased number of active caspase-3 positive cells in DLD-1 tumours treated with the combination therapy (Fig. 7d). Analysis of the serine and glycine levels in the tumours from these mice mirrored the results from the plasma, showing either PH755 treatment or -SG diet lowered intra-tumoral serine and glycine levels (Fig. 7e and Supplementary Figure 7d), although in each case the -SG diet was more effective in lowering intra-tumoral serine and glycine levels that treatment with the PHGDH inhibitor. HCT116 tumours showed a modest further drop in serine and glycine in the combination diet and drug treated mice (Supplementary Figure 7d) but in DLD-1 tumours, the reduction in serine in response to the -SG diet was not further affected by additional PH755 treatment (Fig. 7e). Nevertheless, a further reduction in intra-tumoral glycine in the double-treated mice suggests that flux through the SSP is lower in the double-treated tumours and that the maintenance of the low steady state levels of serine may reflect the decrease in growth (and serine consumption) under these conditions (Fig. 7e).

Our in vitro data showed that complete inhibition of serine availability through serine starvation and PHGDH inhibition led to defects in one-carbon metabolism and a global inhibition of translation that correlated with a failure to induce an ATF-4 response. To examine these responses to dietary serine/glycine starvation and PHGDH inhibition in vivo, we first examined purine levels in the tumours. As noted in vitro (Supplementary Figure 3c), we did not see any difference in total ATP or GTP levels in tumours from double-treated mice (Supplementary Figure 7e), likely reflecting the decreased proliferation of the double-treated tumour cells. In the methionine cycle, the regeneration of SAM from SAH requires one-carbon units. Interestingly, we noted a clear reduction in the SAM/SAH ratio in tumours from -SG diet mice, which was further reduced in mice on -SG diet plus PH755 (Fig. 7f). These results are consistent with a reduction in one-carbon availability in mice on a -SG diet that is exacerbated in double-treated mice.

To examine the ATF-4 response, we measured the expression of the two ATF-4 targets, PHGDH and PSAT1, in DLD-1 tumours (Supplementary Figure 7f, g). Immunohistochemistry analysis of these tumours revealed that feeding mice with a -SG diet led to a clear induction of PSAT1 – and to a lesser extent PHGDH – in tumours, indicating the induction of an ATF-4 response in vivo. By contrast, treating mice with the PHGDH inhibitor alone did not result in any change in PHGDH or PSAT1 expression, suggesting that only dietary restriction of serine and glycine was effective to deplete serine and glycine intra-tumoral levels enough to lead to an ATF-4 response in vivo. Interestingly, the induction of PSAT1 and PHGDH was equivalent or even more pronounced in the double-treated tumours compared to the tumours from mice fed a -SG diet only, showing that, in vivo, the combination treatment did not reduce available serine sufficiently to compromise the ability of tumour cells to induce an ATF-4 response (Supplementary Figure 7f, g). Taken together, these data show that the inhibition of tumour growth correlate with defects in serine metabolism, rather than inhibition of translation.

## Discussion

This study has explored the efficacy of a combination of serine/glycine depletion with PHGDH inhibition to inhibit cancer cell growth. Our analyses of cell lines and organoids showed that when used at a concentration that effectively inhibited de novo serine synthesis, treatment with PH755 alone had no impact on cell growth. These data suggest that all the cell lines used were able to import exogenous serine efficiently enough to maintain proliferation. These results are in contrast to previously reported PHGDH inhibitors that inhibited growth of PHGDH expressing cells in the presence of extracellular serine and glycine^26–28^. These previously published compounds showed weaker activity than PH755, and in some cases exhibited some additional activities, such as electron transport chain inhibition^26^. Differences in the mode of action of the inhibitors (PH755 is an NAD^+^ competitive inhibitor while others function by disrupting the PHGDH tetramer^27^ or as non-competitive inhibitors^26,28^) or in the selectivity of these compounds at the doses used in cell growth assays may account for the different responses in the presence of serine. Previous studies have also shown that cell lines like MDA-MB-468, which express high levels of PHGDH and have activated SSP, are sensitive to sh-RNA mediated depletion of PHGDH, regardless of the presence of serine in the medium^14,15^, indicating a difference between loss and inhibition of PHGDH. It has been suggested that activation of the SSP is important not only for serine production, but can also contribute to the generation of α-ketoglutarate (through the PSAT1 reaction)^14^ or the redox balance in the cell (as PHGDH consumes NAD^+^)^40^. However, depletion and inhibition of PHGDH is unlikely to affect these two processes differently. It is possible that the PHGDH protein has functions additional to supporting the SSP and that this activity is maintained in the presence of the PHGDH inhibitor. Further analysis will be required to address this point.

Using xenograft models of intestinal cancer, we have provided a proof of concept that limiting the availability of serine and glycine to tumours by targeting the exogenous and endogenous supply can have therapeutic benefit. While we did not see a clear beneficial effect of PH755 treatment alone compared to the -SG diet, the combination showed augmented therapeutic efficacy. These observations show some parallels to other approaches aimed at limiting the supply of NEAAs to cancers that are already in clinical use, such as treatment of patients with ASS1 deficient cancers (so blocking the de novo pathway of arginine synthesis) with ADI-PEG20, to degrade circulating arginine^41^. Consistent with a recent study showing no overt toxicity associated with depletion of PHGDH in adult mice (sparing the brain)^42^, mice fed on a normal diet tolerated treatment with PH755 well, showing that in the presence of exogenous serine, inhibition of the SSP was not detrimental to health. Similarly, PH755 did not affect the growth of cells in serine and glycine containing medium. However, we did not assess long-term treatment with the inhibitor, and studies showing that serine synthesis through the activity of PHGDH is important for the maintenance of nucleotide levels^43^ or haem production in endothelial cells^44^ suggests that there may be some longer term on-target effects that would need careful monitoring. In particular, in mice and humans, PHGDH deficiency leads to severe neurological defects, although PHGDH heterozygous mice are normal, despite a reduction in brain serine levels^45,46^. The lack of obvious neurological symptoms following PH755 treatment possibly reflects an incomplete inhibition of the SSP or a lower requirement for PHGDH in the adult brain. Even the combination of -SG diet and PH755 treatment – which effectively lowered circulating serine levels – did not lead to overt morphological changes in the brain, although more detailed behavioural analysis would be required to rule out any effect on brain function. More generally, the mice on the combination treatment appeared active and healthy. There was no evidence of liver or kidney toxicity although a reduction in intestinal villi length correlated with the weight loss observed in these mice. However, the intestinal crypts appeared to be unaffected, consistent with the stabilisation of weight following a reduction in PH755 dosing, even when left on the -SG diet. It remains possible that long-term serine and glycine limitation will result in unwanted toxicities. Indeed, a recent study showing long-term dietary serine and glycine starvation leads to retinal defects in mice^47^ suggests that shorter term application of these approaches for cancer therapy are likely to be required. However, it is encouraging that we were able to maintain the combined drug and diet treatment for long enough to detect a therapeutic benefit without any acute adverse effects. Further studies to establish the optimal effective dose and dosing regime, to balance modulation of serine availability to the tumour with weight loss, will likely lead to even better responses.

The inhibition of tumour growth in response to -SG diet plus PHGDH inhibition shown here are consistent with a recent study showing that depletion of PSAT1 (the second step in the SSP) in liver cancers reduced tumour growth only in a -SG diet^25^. In vitro, we show that removal of extracellular serine and glycine availability combined with inhibition of PHGDH effectively depleted intracellular serine levels and blocked glutathione and nucleotide synthesis in cultured cells, consistent with defects in one-carbon metabolism. Inactivation of mTORC1 in response to amino acid starvation results in a general inhibition of translation and increased autophagy^48^. This response is often coupled with the selective activation of ATF-4 translation, which mediates a protective response including the upregulation of expression of amino acid transporters and de novo synthesis pathways^35,49^. While we also saw induction of an ATF-4 response in cells starved of serine and glycine, we found that the dramatic drop in serine availability seen in response to both serine and glycine starvation and PHGDH inhibition in vitro led to a more profound inhibition of translation that prevented the induction of the protective ATF-4 response. This defect was reflected in the almost complete growth inhibition seen in cells that were grown in both adherent 2D conditions or as organoids following serine and glycine starvation plus PHGDH inhibition. While we were able to find evidence of a defect in one-carbon availability in tumours from double-treated mice, we did not see a loss of an ATF-4 response. It remains possible that synthesis of other proteins may be retarded in double-treated mice, but the maintenance of the ATF-4 response likely reflects the retention of some serine availability to the tumour cell (potentially from surrounding stromal cells). These results highlight that caution needs to be applied to translating in vitro results – where complete blocks to metabolic pathways can be achieved – to in vivo situations – where these pathways may only be partially inhibited. However, the retention of an ATF-4 response may explain why the combination therapy – which strongly inhibited normal cell growth in vitro – was not highly toxic to normal cells in mice.

While our work supports a role for defects in one-carbon metabolism in the success of the combination treatment, other responses to lack of serine availability may also contribute. For example, a recent study has shown that inhibition of tumour growth in -SG fed mice may reflects the production of toxic deoxysphingolipids^24^. While the combination of PH755 with a serine and glycine depleted diet could be beneficial in many cancer types, tumours arising in an environment naturally low in serine could benefit from PH755 treatment without the requirement for additional dietary intervention^23^. As shown recently, treatment with PH755 was effective in limiting serine synthesis and tumour growth of metastases in the brain, where environmental serine and glycine levels are low^31^. Furthermore, tumours carrying other genetic alterations that lead to resistance to dietary serine depletion, such as p53 or KRAS mutations, may be sensitive to the combination therapy described here. Stratification of tumours to account for intrinsic vulnerability to serine limitation and extrinsic limitation of serine levels is likely to further improve the efficacy of this approach.

## Methods

### Cell culture

All the human cell lines used in this study were obtained from the Crick Cell Services. All cell lines underwent routine quality control, which included mycoplasma detection, STR profiling and species identification for validation. Cells were cultured at 37 °C in a humidified atmosphere of 5% CO_2_. HT-29, SW48, SW480, SW620, CACO2, HCT116, RKO, VACO5 and MDA-MB-468 cells were cultured in DMEM (Gibco, 41966) supplemented with 10% FBS; DLD-1, HCT-15 and SW1417 cells were cultured in RPMI 1640 medium (Gibco, 21875) supplemented with 10% FBS and LoVo and CL-34 cells were cultured in DMEM/F12 (Gibco, 11320) supplemented with 10% FBS.

#### Serine and glycine deprivation

For all serine and glycine-deprivation experiments, cells were cultured in MEM (Gibco, 21090) supplemented with 10% dialysed FBS (Hyclone, Thermo Scientific), 1% penicillin–streptomycin, d-glucose (5 mM), sodium pyruvate (65 µM), 1X MEM vitamin solution (Gibco, 11120), l-Glutamine (2 mM), l-Proline (0.15 mM), l-Alanine (0.15 mM), l-Aspartic acid (0.15 mM), l-Glutamic acid (0.15 mM) and l-Asparagine (0.34 mM) (-SG media). The complete medium (CM) corresponds to the previously described medium supplemented with 0.4 mM l-Serine and 0.4 mM l-Glycine.

#### Growth curves

Cells (2 × 10^4^ to 3 × 10^4^ cells/well depending on the cell lines) were plated in 24-well plates in their regular medium. The next day, after being washed with PBS, cells were transferred to -SG medium or CM and treated with 10 µM PH755 (RAZE Therapeutics) diluted in DMSO or DMSO alone. For the counting step, cells were trypsinized, suspended in PBS-EDTA and counted with a CASY Model TT Cell Counter (Innovatis, Roche Applied Science). Relative cell number at each time point was calculated based on the number of cells measured before the medium change. For the growth curve experiment with formate and glycine supplementation, HT-29, HCT116 and DLD-1 cells were seeded in 24-well plates (2 × 10^4^ cells/well). Sodium formate (Fluka Analytical, 71540) (1 mM) and/or glycine (0.4 mM) were diluted in -SG medium + 10 µM PH755 and medium was refreshed every two days.

#### Organoids

Crypts were isolated from adenomatous small intestine tissue derived from *Vil1-creER;Apc*^*fl/fl*^ and *Vil1-creER;Apc*^*fl/fl*^*;Kras*^*G12D/+*^ mice as previously described^17^. The generation of the *Apc5* organoid bearing an *Apc* truncating mutation using CRISPR/Cas9 technology and isolation of normal organoids derived from the proximal part of healthy small intestine from *Villin*-CreERT2 mouse were performed as previously described^50–52^. The generation of the four patient-derived colorectal organoids used in this study has been described previously^32^. Cancer organoids from mice were cultured in tumour organoid medium (CM) composed of Advanced DMEM/F12 (Gibco, 12634) supplemented with 1% penicillin–streptomycin solution, 0.1% BSA, 2 mM l-glutamine, 10 mM Hepes, 50 ng/mL EGF (PeproTech AF-100-15), 100 ng/mL Noggin (PeproTech 250-38), 500 ng/mL Spondin (PeproTech 120-38), 1X N-2 Supplement (ThermoFisher 17502048) and 1X B-27 Supplement (ThermoFisher 17504044). The -SG medium corresponds to the previously described medium without serine and glycine. Normal organoids from mice were grown in normal organoid medium, a modification of tumour organoid medium that was supplemented with 100 ng/mL Wnt-3a (R&D systems, 5036-WN), 1 mM *N*-Acetyl-l-cysteine (Sigma, A7250), 10 μM Y-27632 (Sigma, Y0503) and 4 mM Nicotinamide (Sigma, N0636). Human organoids were grown in human organoid medium, a second modification of tumour organoid medium that was supplemented with 10 nM FGF-basic (PeproTech, 100-18B), 100 ng/mL Wnt-3a (R&D systems, 5036-WN), 1 μM Prostaglandin E2 (Tocris, 2296), 4 mM Nicotinamide (Sigma, N0636), 20 ng/mL HGF (PeproTech, 100-39), 10 nM FGF-10 (PeproTech, 100-26), 10 nM Gastrin I (Sigma, G9145), 10 μM Y-27632 (Sigma, Y0503), 0.5 μM A 83-01 (Tocris, 2939) and 5 μM SB 202190 (Sigma, S7067). For the splitting step, organoids were harvested through mechanical pipetting using TrypLE (Gibco, A12177-01), incubated for 10 min at 37 °C, diluted three times in volume in ice-cold 1X HBSS (Gibco, 14175-053) and spun down at 270×*g* for 5 min at 4 °C. Pellet was then resuspended in growth factor reduced Matrigel (Corning, 356231) and plated in 24-well plates. Matrigel was then incubated for 15 min at 37 °C and 1 mL of the CM described above was added. The next day, organoids were washed with PBS and the medium was replaced with CM or -SG medium supplemented or not with 10 µM PH755 and allowed to grow. Pictures were regularly taken with a light microscope (Zeiss, Axiovert 40 CFL) using Infinity Capture (version 6.5.4) and organoid diameter was measured using ImageJ software.

### Generation of PHGDH KO cells

pLentiCRISPRv2 vector containing the following guide RNA: TGGACGAAGGCGCCCTGCTC was purchased from Genscript to target *PHGDH*. HEK293T cells were transfected with this lentiviral plasmid together with psPAX2 (Addgene, 12260) and VSV.G (Addgene, 14888) using jetPRIME reagent (Polyplus transfection). After 24 h incubation, medium was changed and 48 h later, the viral particle containing-medium was filtered (0.45 mm) and mixed with polybrene (4 µg/ml, Sigma-Aldrich). The medium containing lentiviruses was incubated with the target cells for 24 h. HT-29 and DLD-1 cells were then selected with puromycin (Sigma-Aldrich) for 3 weeks and analysed for loss of PHGDH expression.

### ATF-4 siRNA transfection

The siRNA used to downregulate human ATF-4 and the non-targeting siRNA control were purchased from Dharmacon (siGENOME SMART pool siRNA). Cells were transfected with siRNA using Lullaby transfection reagent (OZ Biosciences) following the manufacturer’s instructions.

### BrdU/7-AAD staining

HCT116 and DLD-1 cells were grown for 48 h in -SG medium or CM and treated with 10 μM PH755 diluted in DMSO or DMSO alone. To determine the percentage of bromodeoxyuridine (BrdU) positive cells, 10 μM BrdU was then added to culture media for an additional 5 h while for cell cycle analysis, 10 μM BrdU was added for only 30 min. Cells were then harvested, fixed and stained with APC anti-BrdU antibody (and 7-AAD for cell cycle analysis) using the APC BrdU Flow kit (BD Pharmingen, Cat no: 552598) following the manufacturer’s instructions. Fluorescence was acquired with FACSdiva on a Fortessa flow Cytometer and the analysis performed using FlowJo (version 10.5.2).

### Western blot

Protein lysates were processed in RIPA-buffer (Millipore) supplemented with phosphatase inhibitor cocktail (Thermo Fisher Scientific) and complete protease inhibitors (Roche). Lysates were separated using precast NuPAGE 4–12% Bis-Tris Protein gels (Invitrogen) and transferred to nitrocellulose membranes. Following incubation with primary antibodies, appropriate secondary antibodies were used to detect the proteins. Western blots were scanned using the LI-COR Odyssey infrared scanner (imaged using LI-COR Image Studio Lite software version 5.2.5) or visualised using ECL chemiluminescence detection kits (Pierce). Primary antibodies used were as follows: PHGDH (13428), ATF-4 (11815), Phospho-eIF2α (Ser51) (3398), Phospho-p70S6 kinase (Thr389) (9234), p70S6 kinase (9202), c-Myc (5605), HIF1α (14179), Caspase-3 (9662), Cleaved Caspase-3 (Asp175) (9661), beta-Actin (4970) from Cell Signaling Technology; GCN2 (sc-374609), eIF2α (sc-133132), p53 (sc-126), Vinculin (sc-73614) from Santa Cruz Biotechnology; PSAT (ab96136), PSPH (ab96414), Phospho-GCN2 (Thr899) (ab75836) from Abcam; ASNS (HPA029318) from Atlas Antibodies; Puromycin (MABE343) from Sigma-Aldrich. All primary antibodies were diluted at 1:1000 dilution except puromycin antibody (1:20,000). Uncropped and unprocessed scans of the most important blots are supplied in the Source Data file.

### Protein synthesis and degradation

Cells were grown for 24 h in -SG medium or CM and treated with 10 μM PH755 diluted in DMSO or DMSO alone. To evaluate protein synthesis, puromycin (final concentration: 90 μM) was added to each well 10 min prior harvesting the cells for western blot analysis, except in the negative control well. Where indicated, cells grown in CM medium were treated with cycloheximide (10 μg/mL) for the last 5 h providing a control for translation inhibition. Incorporation of puromycin into newly synthesised proteins was assessed by western blot using an anti-puromycin antibody (Sigma-Aldrich, MABE343). To assess the accumulation of short-lived proteins in response to proteasome inhibition, cells grown in -SG medium or CM plus or minus 10 μM PH755 for 24 h were treated for the last 6 h with the proteasome inhibitor MG-132 (10 μM) before harvesting the cells for western blot analysis.

### qPCR

HT-29, HCT116 and DLD-1 cells were grown for 6 h or 24 h in -SG medium or CM and treated with 10 μM PH755 diluted in DMSO or DMSO alone. Total RNA was extracted using RNeasy Mini kit (Qiagen, Cat No.: 74104) performing on-column digestion of DNA (Qiagen, RNase-Free DNase Set, Cat No.: 79254) and reverse transcribed using the High-Capacity cDNA Reverse Transcription kit (Thermofisher, Cat No.: 4368814) according to the manufacturer’s instructions. qPCR was performed using PrimeTime Gene Expression Master Mix (IDT, Cat No: 1055771) with the primers listed in Supplementary Table 1. The QuantStudio 7 Flex Real-Time PCR System (software v1.3) was used for all reactions. Gene expression was normalised to *ACTB* (β-actin) housekeeper gene, analysed according to Pfaffl method^53^ and expressed as relative units compared to the cells grown in CM for 6 h.

### Liquid chromatography–mass spectrometry

HT-29 cells (2.4 × 10^5^), HCT116 cells (1.8 × 10^5^), DLD-1 cells (1.8 × 10^5^) and MDA-MB-468 cells (2.4 × 10^5^) were plated in six-well plates in their regular medium. Duplicate plates were used for cell counting to normalise LC-MS analysis based on cell number. After 16 h, cells were washed with PBS and transferred to CM or -SG medium supplemented or not with 10 µM PH755 for 24 h. In all, 6 h before metabolite extraction, medium was replaced with CM or -SG medium ±10 µM PH755 with glucose substituted for 10 mM U-[^13^C]-glucose (Cambridge Isotopes). For short-term experiments, cells were moved to the previously described medium with glucose substituted for 10 mM U-[^13^C]-glucose for only 3 h or 6 h before metabolite extraction. For measurement of glycine conversion into serine during rescue experiment, cells were grown for 24 h in -SG medium supplemented with 10 µM PH755, 1 mM sodium formate and 0.4 mM glycine. This medium was then replaced with matched medium with glycine substituted for 0.4 mM ^13^C_2_^15^N_1_-glycine for 1 h before metabolite extraction. For half of the samples, a pulse of 1 mM unlabelled serine was added to the medium 1 min before metabolite extraction to allow labelled serine to accumulate. Cells were then washed with PBS and metabolites were extracted using ice-cold extraction buffer composed of methanol, acetonitrile and H_2_O in the following ratio 50:30:20. For LC-MS analysis on tumour samples, tissue was homogenised (20–40 mg tissue/mL of the previously described extraction buffer) using the Precellys 24 homogenizer (Bertin Instruments). Samples were spun (16,000×*g*/10 min/0 °C) and the supernatant collected to be centrifuged again (16,000×*g*/10 min/0 °C). Supernatant were then collected for LC-MS analysis. For LC-MS analysis on mice plasma, plasma was diluted 20–50 fold with the same extraction buffer, vortexed for 30 s and centrifuged (16,000×*g*/10 min/0 °C). Supernatant were then collected for analysis. Absolute levels of serine and glycine in plasma was determined using 8-point calibration curves (from 2.5 to 800 µM) with ^13^C_3_^15^N_1_-serine and ^13^C_2_^15^N_1_-glycine diluted in plasma. LC-MS analysis was performed as previously described^9^. Data were recorded using Xcalibur 4.0.27.19 software (Thermo Scientific). Verification of qualitative peak assignments was done with Qual Browser from Xcalibur 4.0.27.19 and metabolite peak integration was performed using TraceFinder 4.1.

### In vivo experiments

All animal studies were conducted in compliance with UK Home Office approved project licences (Animals (Scientific Procedures) Act 1986 and the EU Directive 2010). Animal experiments were subject to ethical review by the Francis Crick Animal Welfare and Ethical Review Body and carried out under UK Home Office project license P319AE968 or by the CRUK Beatson Institute (reviewed and approved by the University of Glasgow and UK Home Office) and carried out under UK Home Office project license 70/8645. Mice (3–5 per cage) were allowed access to food and water ad libitum and were kept in a 12-h day/night cycle starting at 7:00 until 19:00. Rooms were kept at 21 °C at 55% humidity. Mice were allowed to acclimatise for at least 1 week prior to the experiment. They were then randomly assigned to experimental groups. The experimental diets used in this study (control diet and -SG diet) were previously described as “Diet 1-Control” and “Diet 1-SG-free”^17^. Briefly, the control diet contains all essential amino acids as well as serine, glycine, glutamine, arginine, cystine and tyrosine. The -SG diet is the same as the control diet but is deprived of serine and glycine, which are compensated by a proportionally increased level of the other amino acids to reach the same total amino acid content.

#### Xenograft experiments

CD-1 female nude mice (obtained from Charles River, 7–9 weeks old) received unilateral subcutaneous injections of 100 µl of HCT116 cells (2 × 10^6^ cells) or 100 µl of DLD-1 cells (4 × 10^6^ cells) suspended in PBS. Mice were placed on experimental diets (control or -SG) 10 days (for HCT116 xenograft experiment) or 2 days (for DLD-1 xenograft experiment) after tumour injections. In all, 4 days (for HCT116 xenograft experiment) or 2 days (for DLD-1 xenograft experiment) after the diet change, mice were treated either with vehicle (0.5% methylcellulose (Sigma, H7509), 0.5% Tween-80 (Sigma, P8192)) or PH755 (obtained from Raze Therapeutics) prepared in vehicle once daily by oral gavage. The starting dosage of PH755 was 100 mg/kg and was subsequently lowered to 75 mg/kg or 50 mg/kg as indicated in the figure legends. Subcutaneous growth was measured two to three times a week by caliper and the following formula: length × width^2^/2 was used to calculate tumour volume.

#### C57BL/6J experiment

C57BL/6J male mice (obtained from Charles River, 14 weeks old) were placed on experimental diets (control or -SG) 2 days before starting the treatment with PH755 or its vehicle. Mice were treated once daily by oral gavage with PH755 or its vehicle for 20 days. The starting dosage of PH755 was 75 mg/kg and was subsequently lowered to 50 mg/kg to maintain weigh loss below 20% of the initial body weight.

### Immunohistochemistry

All tissues were fixed in 10% neutral buffered formalin and were embedded in paraffin. For PHGDH and PSAT1 staining, the slides were de-paraffinised in xylene and rehydrated using a series of graded industrial methylated spirits solutions and distilled water. Antigen retrieval was performed for 23 min in the microwave using pH 6 0.1 M citrate buffer. Endogenous peroxidase blocking was performed using 1.6% H_2_O_2_ for 10 min at room temperature and protein blocking was performed using 2.5% Normal Horse Serum (MP-7401, Vector) overnight at 4 °C. Primary antibody was diluted at 1:1000 for PHGDH antibody (HPA021241, Sigma-Aldrich) and at 1:500 for PSAT1 antibody (PA5-22124, Thermofisher) in 1% BSA, and incubated for 1 h at room temperature. HRP Horse Anti-Rabbit IgG Polymer (MP-7401, Vector) was incubated for 30 min at room temperature. 3,3-diaminobenzidine (DAB) chromogen (SK-4100, Vector) was incubated for 10 min at room temperature. The slides were counterstained with Harris Heamatoxilin, dehydrated, cleared and mounted in a Sakura Tissue-Tek Prisma® auto stainer. For PHGDH and PSAT1 staining intensity quantification, a minimum of three fields per tumour were quantified as described in Crowe et al.^54^ with ImageJ. Active Caspase-3 immunohistochemistry was performed on the Discovery Ultra Ventana platform (from Roche). Antigen retrieval was obtained with Cell Conditioning 1 (CC1) from Ventana Medical Systems. Primary antibody (AF835, R&D Systems) was diluted at 1:1250 and incubated for 60 min. For Active Caspase-3 staining, a minimum of three fields per tumour were quantified with the positive cell detection algorithm from QuPath (version 0.1.2). All slides were scanned with the ZEISS Axio Scan.Z1 slide scanner and images were generated through ZEISS ZEN 2.6 (blue edition) software. For gut rolls, immunohistochemistry was performed on Bond Rx Autostainer from Leica Biosystems using Leica Bond Intense R staining kit. Slides were de-paraffinised with Bond Dewax at 72 °C for 30 min and antigen retrieval was achieved with ER2 at 100 °C for 20 min. Primary antibody (Ki67 SP6, ab16667, Abcam) was diluted at 1/100 and incubated for 35 min. For villus length measurement, villi from the same area of the small intestine (at least 15 per mouse) were measured from the crypt/villus junction to the villus tip, using ImageJ.

### Brain sampling and pathological examination

C57BL/6J mice were culled using carbon dioxide asphyxiation to avoid physical trauma to the brain. Mice were immediately dissected and haired skin and soft tissue were removed from the cranial surface. Incisions throughout the parietal and frontal sutures were performed to allow fast penetration of the fixative solution into the brain parenchyma. The head was immersed in 250 mL of 10% neutral buffered formalin and fixed for 2 weeks. After complete fixation, the brains were removed from the skull and trimmed using a mouse brain matrix (BSMYS001-1; Zivic Instruments, Pittsburgh, PA). Four coronal sections were obtained at the level of the pyriform cortex, caudal diencephalon, caudal mesencephalon and rostral cerebellum. Tissue samples were routinely processed for paraffin embedding, sectioned at 4 μm, and stained with haematoxylin and eosin. The histopathologic criteria described in the INHAND diagnostic scheme was consulted to identify possible microscopic changes^55^. Histopathological examination of brains was performed by a board-certified veterinary pathologist.

### Blood biochemical marker assays

Plasma ALT and AST activities were measured using Alanine Transaminase Activity Assay Kit (Abcam, Cat No.: ab105134) and AST Activity Assay Kit (Sigma-Aldrich, Cat No.: MAK055-1KT) respectively, according to the manufacturer’s instructions.

### Statistical analyses

All data are expressed as mean ± SEM and each statistical analysis is detailed in the figure legend. Data were collected in Excel (version 16.16.26) and all statistical analyses were performed using GraphPad Prism 8 (version 8.3.1) software. Unpaired Student’s *t* test was performed to compare two groups to each other. If the variance between the two groups was unequal, a Welch’s correction was applied. To compare more than two groups, statistical significance was determined using one-way ANOVA with Tukey’s multiple comparison test. For tumour volume and body weight analyses, two-way ANOVA plus Tukey’s post hoc test were performed. *p* value below 0.05 was considered statistically significant. Significance is indicated as follows: *p < 0.05, **p < 0.01, ***p < 0.001, ****p < 0.0001, ns: no significance. All measurements were taken from distinct samples. Sample sizes were based on standard protocols in the field and the metabolic samples were assigned in a random order before analysis. Mice were randomly assigned to a treatment and the identity of each mouse was blinded when measurements were collected.

### Reporting summary

Further information on research design is available in the Nature Research Reporting Summary linked to this article.

## Supplementary information

Supplementary Information

Reporting summary

## Data Availability

Source data are provided with this paper. All the other data supporting the findings of this study are available within the article and its supplementary information files and from the corresponding author upon reasonable request. Source data are provided with this paper.

## Source data

Source Data
