# Supplementary material for: Serine synthesis pathway inhibition cooperates with dietary serine and glycine limitation for cancer therapy
Source: Nat Commun. 2021 Jan 14;12:366. doi: 10.1038/s41467-020-20223-y (PMC7809039; doi:10.1038/s41467-020-20223-y)
Supplement: Supplementary file 1 — Supplementary Information [file 41467_2020_20223_MOESM1_ESM.pdf]

Supplementary Information

Serine synthesis pathway inhibition cooperates with dietary serine and glycine limitation for cancer therapy

Tajan et al.

Supplementary Table 1. Primers used in the study for qPCR.

Gene name	Primer 1	Primer 2	Probe
ASNS	AGTACAGTATCCTCTCAGACA	TCACTTCCAATATGATCTGCCA	TTCTAGCAGCCAGTAAATCGGGGC
ATF4	AGGTGTCTTTGTCGGTTACAG	CGTATTAGGGGCAGCAGTG	CCATGGCGCTTCTCACGGC
PHGDH	CACTGAGGCTGTTCCCATT	GTCATCAACGCAGCTGAGAA	CCAGATCCACATTGTCCACACCTG
PSAT	TCATCACGGACAATCACCAC	GTCCTCAAACTTCCTGTCCAA	AGAGCCAACATTCTTCTGGGCACC
PSPH	CATGATTGGAGATGGTGCCA	TTATCCTTGACTTGTTGCCTGA	TGTCCTCCTGCTGATGCTTTCATTGG
ACTB	CCTTGCACATGCCGGAG	ACAGAGCCTCGCCTTTG	TCATCCATGGTGAGCTGGCGG

Supplementary Figures

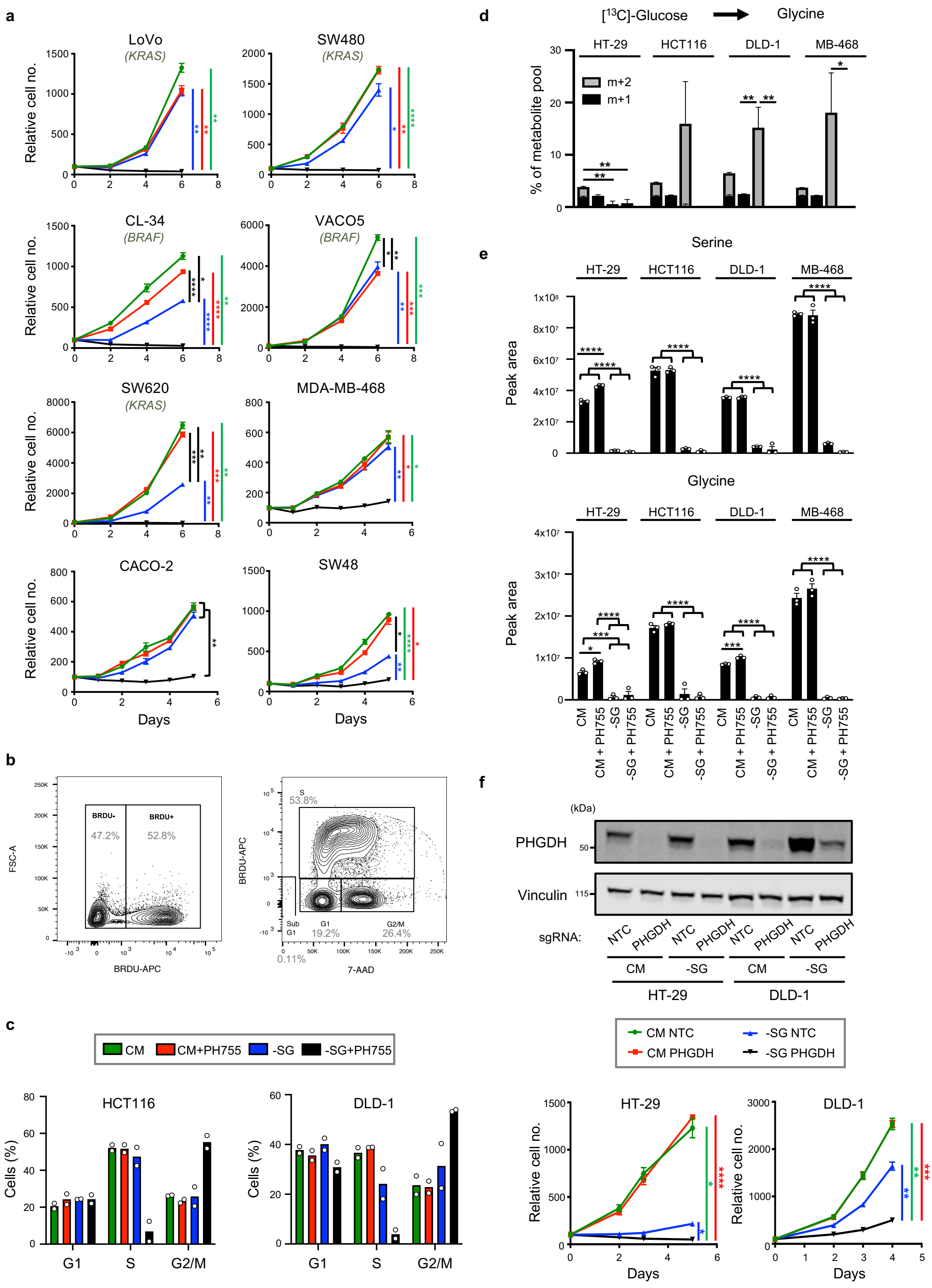

Supplementary Figure 1: Characterisation of cancer cell behaviour following serine/glycine starvation and PHGDH inhibition.

(a). Growth curves of the indicated cell lines grown in complete medium (CM) or equivalent medium lacking serine and glycine (-SG) and treated or not with 10μM PH755. Data are presented as mean ± SEM of triplicate cultures and are representative of at least two independent experiments (* $p < 0.05$, ** $p < 0.01$, *** $p < 0.001$, **** $p < 0.0001$; two-way ANOVA with Tukey's post hoc test).

(b). Gating strategy to determine the percentage of BrdU positive cells (left panel) and the percentage of cells undergoing different phases of the cell cycle (right panel), taking as an example HCT116 cells grown in CM and incubated for 30 minutes with 10μM BrdU.

(c). Cell cycle analysis of HCT116 and DLD-1 cells grown in CM or -SG medium +/- 10μM PH755 for 48 hours followed by a 30 minutes incubation with 10μM BrdU. Data represents mean of two independent experiments.

(d). Intracellular glycine level in HT-29, HCT116, DLD-1 and MDA-MB-468 cells grown in CM or -SG medium +/- 10μM PH755 containing U-[^{13}C]-glucose was measured by LC-MS. Metabolite percentages are represented as mean ± SEM of triplicate cultures and are representative of three independent experiments (* $p < 0.05$, ** $p < 0.01$; one-way ANOVA with Tukey's post hoc test).

(e). Intracellular serine and glycine levels in HT-29, HCT116, DLD-1 and MDA-MB-468 cells grown in CM or -SG medium +/- 10μM PH755 were measured by LC-MS. Data are presented as mean ± SEM of triplicate cultures and are representative of three independent experiments (*$p < 0.05$, ***$p < 0.001$, ****$p < 0.0001$; one-way ANOVA with Tukey's post hoc test).

(f). Top panel: HT-29 and DLD-1 cells infected with Cas9/PHGDH single guide RNA (sgRNA) were cultured in CM or -SG medium for 24 hours. Western blot shows efficient PHGDH depletion in these cells. Membrane was reprobed with vinculin as a loading control. Bottom panel: Growth curves of HT-29 and DLD-1 cells infected with Cas9/PHGDH sgRNA (PHGDH) grown in CM or in -SG medium. Data are presented as mean ± SEM of triplicate cultures and are representative of three independent experiments (* $p < 0.05$, ** $p < 0.01$, *** $p < 0.001$, **** $p < 0.0001$; two-way ANOVA with Tukey's post hoc test).

Source data are provided as a Source Data file.

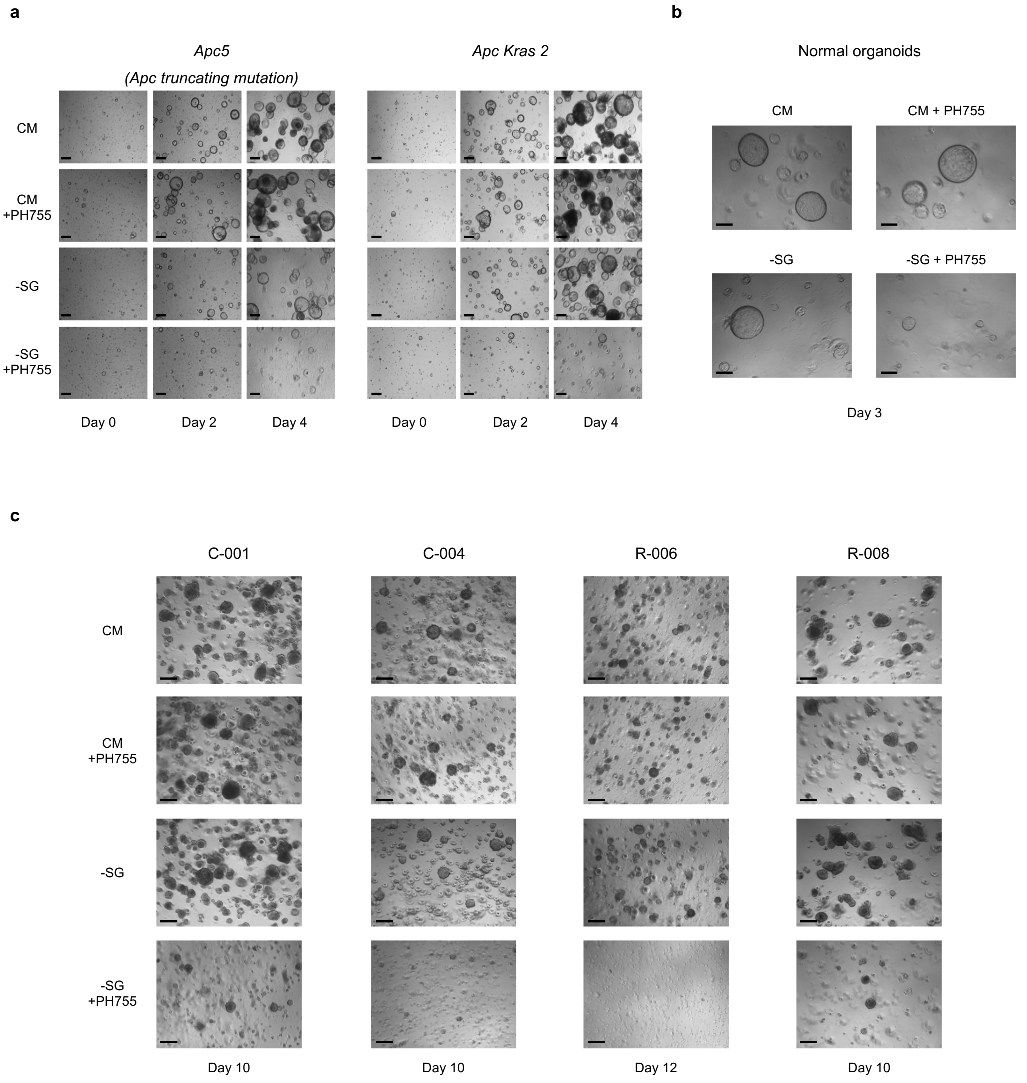

Supplementary Figure 2: PHGDH inhibition cooperates with serine/glycine depletion to impede the growth of mouse and human intestinal organoids.

(a). Intestinal organoids with *Apc* truncation (*Apc5*) or derived from *Villin-CreER;Apc*$^{fl/fl}$*;Kras*$^{G12D/+}$ mice (*Apc Kras 2*) were grown for 4 days in tumour organoid medium with (CM) or without (-SG) serine and glycine supplemented or not with 10µM PH755. Representative pictures of the organoids from at least 2 independent experiments are shown before (day 0), 2 days and 4 days after medium change. Scale bar represents 200 µm.

(b). Normal organoids derived from the proximal part of healthy small intestine from a *Villin-CreERT2* mouse were grown in normal organoid medium (containing Wnt-3a) with (CM) or without (-SG) serine and glycine supplemented or not with 10µM PH755. Representative

pictures of the organoids from 3 independent experiments are shown 3 days after medium change. Scale bar represents 200 μm.

(c). Four patient-derived colorectal organoids were grown in human organoid medium with (CM) or without (-SG) serine and glycine supplemented or not with 10μM PH755. Representative pictures of the organoids from at least 2 independent experiments are shown 10 to 12 days after medium change. Scale bar represents 200 μm.

Source data are provided as a Source Data file.

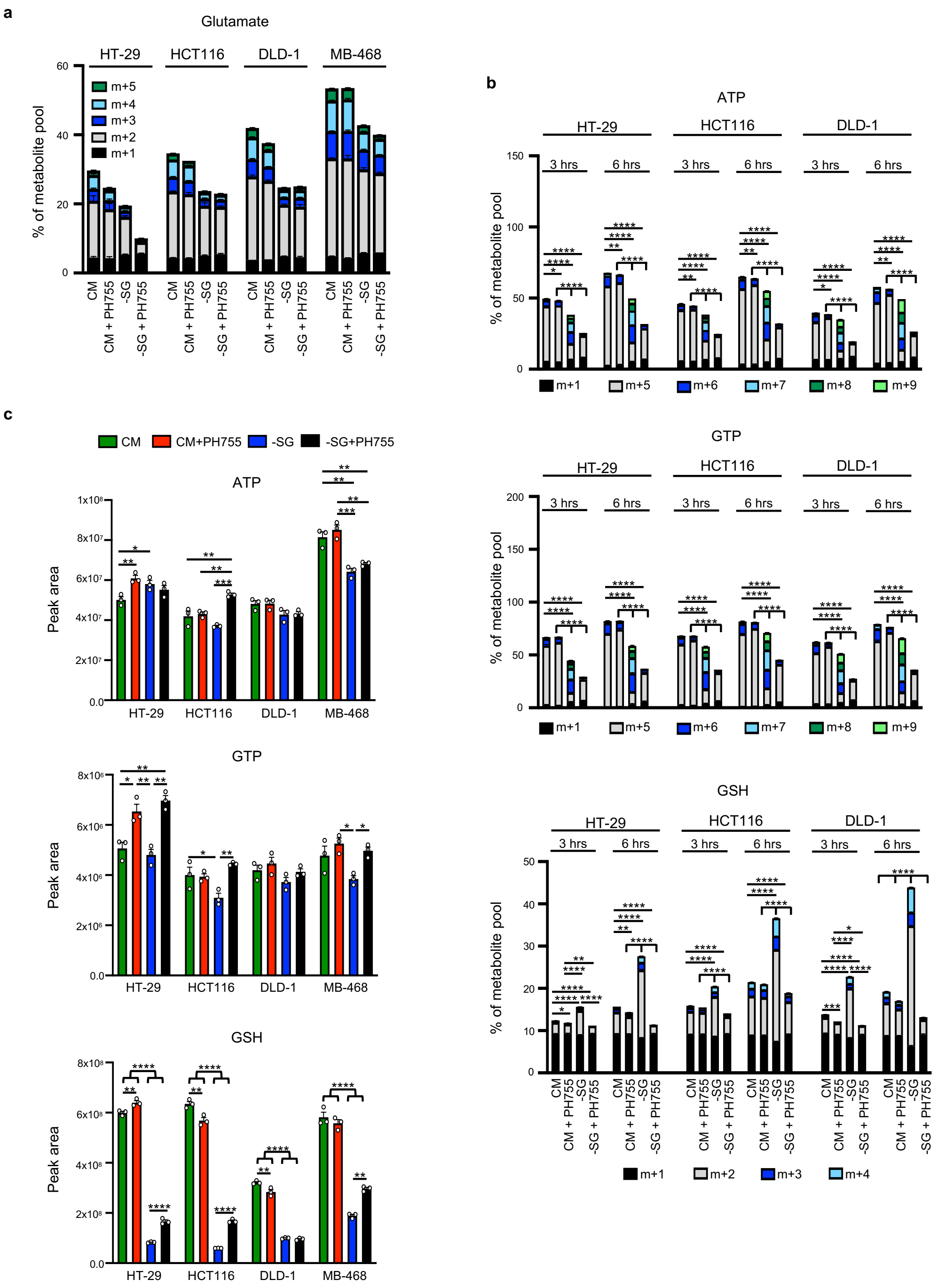

Supplementary Figure 3: Metabolic characterisation of cancer cells grown in serine/glycine free medium in presence of the PHGDH inhibitor.

(a). Intracellular glutamate levels in cells grown in CM or -SG medium +/- 10µM PH755 containing U-[^{13}C]-glucose were measured by LC-MS. Metabolite percentages are represented as mean ± SEM of triplicate wells and are representative of three independent experiments.

(b). Intracellular ATP, GTP and GSH levels in HT-29, HCT116 and DLD-1 cells grown in CM or -SG medium +/- 10μM PH755 containing U-[^{13}C]-glucose for 3 hours or 6 hours were measured by LC-MS. Metabolite percentages are represented as mean ± SEM of triplicate cultures and are representative of two independent experiments (* $p < 0.05$, ** $p < 0.01$, ***$p < 0.001$, **** $p < 0.0001$; one-way ANOVA with Tukey's post hoc test).

(c). Total levels of ATP, GTP and GSH in cells grown in CM or -SG medium +/- 10μM PH755 were measured by LC-MS. Data are presented as mean ± SEM of triplicate cultures and are representative of three independent experiments (* $p < 0.05$, ** $p < 0.01$, ***$p < 0.001$, **** $p < 0.0001$; one-way ANOVA with Tukey's post hoc test).

Source data are provided as a Source Data file.

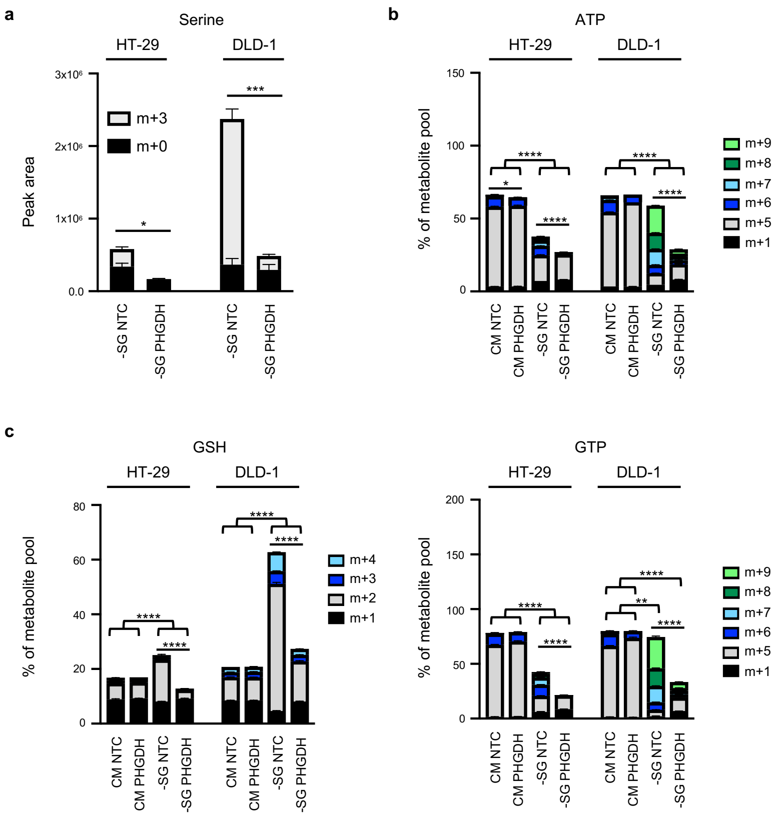

Supplementary Figure 4: Genetic deletion of PHGDH inhibits purine and glutathione synthesis in cells grown in serine/glycine free medium.

(a-c). HT-29 and DLD-1 cells infected with Cas9/PHGDH sgRNA (PHGDH) were grown in CM or in -SG medium in presence of U-[^{13}C]-glucose. Serine (a), ATP (b), GTP (b) and GSH (c) levels were measured by LC-MS. Data are presented as mean ± SEM of triplicate cultures and are representative of two independent experiments (* $p < 0.05$, ** $p < 0.01$, *** $p < 0.001$, **** $p < 0.0001$; (a) unpaired two-sided Student *t* test, (b-c) one-way ANOVA with Tukey's post hoc test).

Source data are provided as a Source Data file.

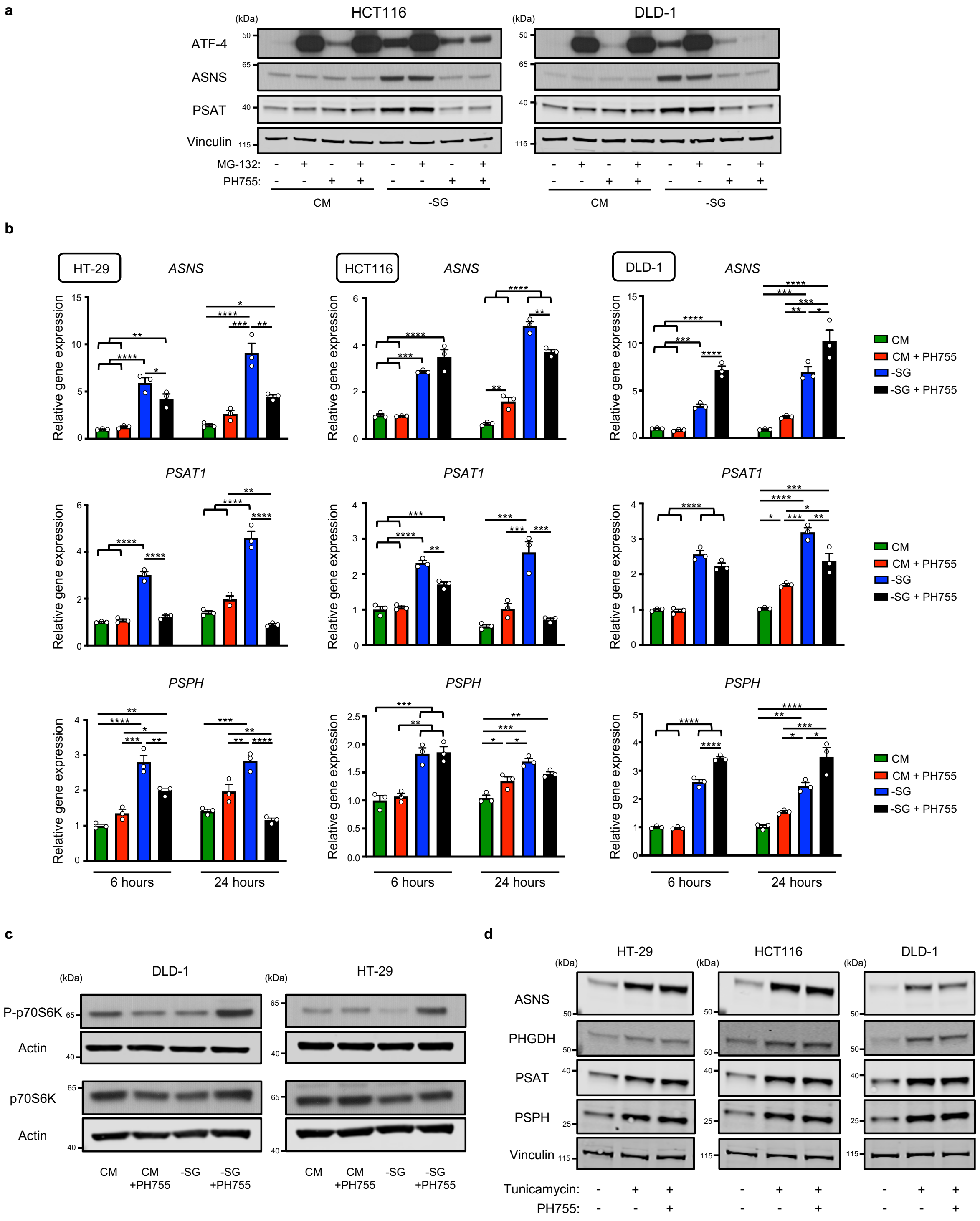

Supplementary Figure 5: ATF-4 response and mTORC1 activation in cancer cells grown in serine/glycine free medium in presence of the PHGDH inhibitor.

(a). HCT116 and DLD-1 cells were grown in CM or -SG medium supplemented or not with 10µM PH755 for 24 hours. When indicated, cells were treated with 10µM MG-132, a proteasome inhibitor, 6 hours before harvesting the cells. Western blots show the expression of ATF-4 and its targets ASNS and PSAT. Membrane was reprobed with vinculin as a loading control. Data are representative of three independent experiments.

(b). HT-29, HCT116 and DLD-1 cells were grown in CM or -SG medium supplemented or not with 10µM PH755 for 6 hours or 24 hours. Relative gene expression of *ASNS, PSAT1* and *PSPH* were measured by qPCR and normalised to the cells grown in CM for 6 hours. Data are presented as mean ± SEM of triplicate cultures and are representative of two independent experiments (* $p < 0.05$, ** $p < 0.01$, *** $p < 0.001$, **** $p < 0.0001$; one-way ANOVA with Tukey's post hoc test).

(c). Cells were grown in CM or -SG medium supplemented or not with 10µM PH755 for 24 hours. Western blots show Phospho-p70S6K (Thr389) and p70S6K. Membrane was reprobed with actin as a loading control. Data are representative of three independent experiments.

(d). HT-29, HCT116 and DLD-1 cells were grown in CM for 24 hours. When indicated, cells were treated with the ER stress inducer, tunicamycin (5 µg/mL) or 10µM PH755. Western blots show the expression of the ATF-4 targets ASNS, PHGDH, PSAT and PSPH. Membrane was reprobed with vinculin as a loading control. Data are representative of two independent experiments.

Source data are provided as a Source Data file.

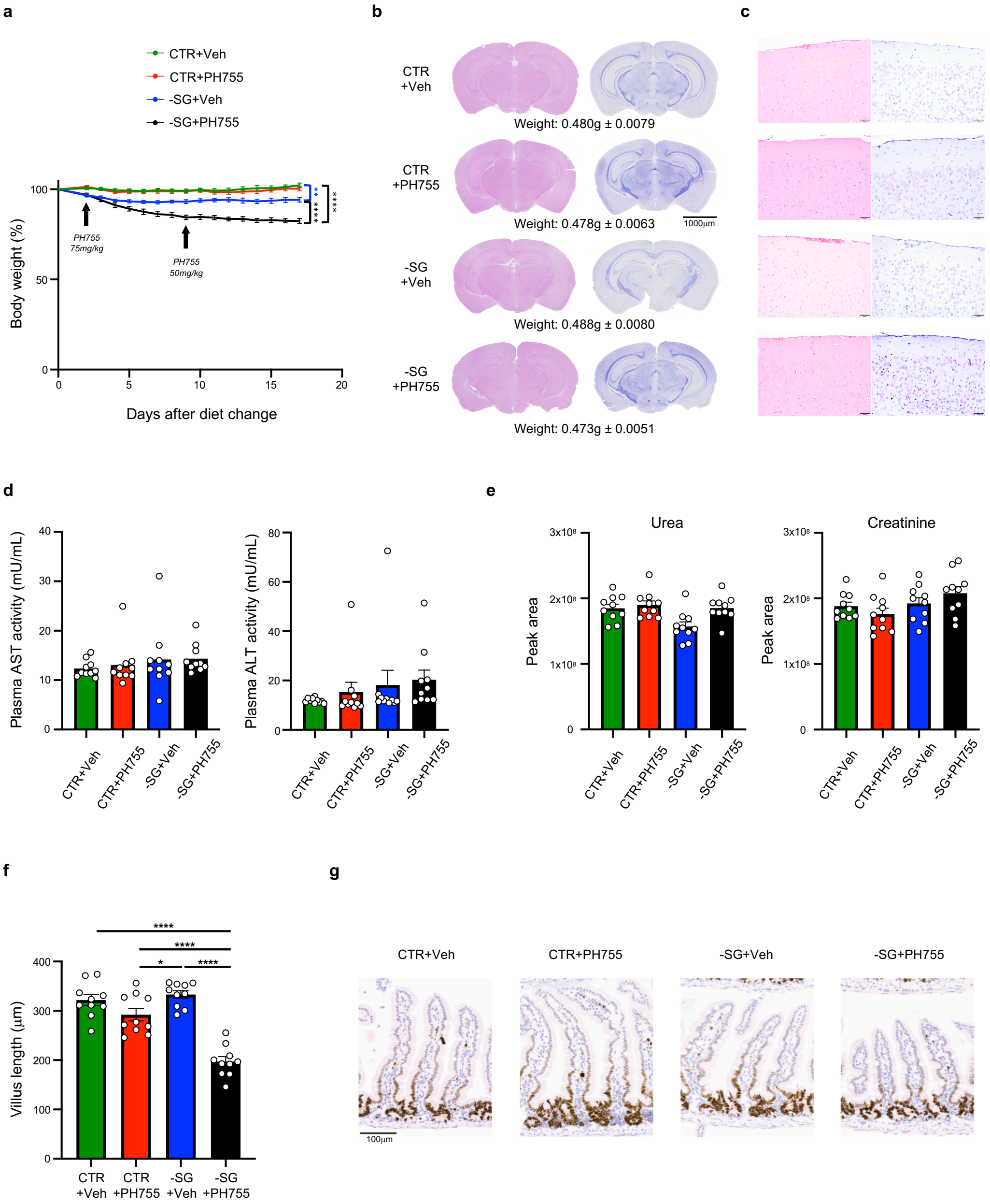

Supplementary Figure 6: Impact of -SG diet and PHGDH inhibition in normal C57Bl6/J mice.

(a). The weight of C57BL/6J mice fed a control diet (CTR) or an equivalent diet lacking serine and glycine (-SG) and treated with vehicle (Veh) or PH755 was recorded at regular intervals. Percentage of body weight was calculated from the initial weight taken the day of the diet change. Arrows show the starting day of the indicated treatment. Data are presented as mean ± SEM (n=10 mice per group). (** $p < 0.01$, **** $p < 0.0001$; two-way ANOVA with Tukey's post hoc test).

(b). Low-power magnifications of transverse sections obtained at the level of the caudal diencephalon and rostral mesencephalon from C57BL/6J mice fed a control diet (CTR) or an equivalent diet lacking serine and glycine (-SG) and treated with vehicle (Veh) or PH755 (n=5 mice per group). There is no evidence of degeneration or malacia on haematoxylin and eosin stained sections. Brain weight for each experimental group of mice is shown as mean ± SEM (n=5 mice/group).

(c). High-power magnifications of transverse sections obtained at the level of the cerebral cortex from C57BL/6J mice fed a control diet (CTR) or an equivalent diet lacking serine and glycine (-SG) and treated with vehicle (Veh) or PH755 (n=5 mice per group). Neurons and neuropil are morphologically unremarkable. Scale bar represents 50 μm.

(d). Plasma was taken at time of sacrifice from C57BL/6J fed a control diet (CTR) or an equivalent diet lacking serine and glycine (-SG) and treated with vehicle (Veh) or PH755 for 20 days. AST and ALT activities in plasma were measured with commercial kits. Data are presented as mean ± SEM (n=10 mice per group).

(e). Plasma was taken at time of sacrifice from C57BL/6J mice fed a control diet (CTR) or an equivalent diet lacking serine and glycine (-SG) and treated with vehicle (Veh) or PH755 for 20 days. LC-MS analysis was performed to evaluate urea and creatinine content. Data are presented as mean ± SEM (n=10 mice per group).

(f). Quantification of villus length from C57BL/6J mice fed a control diet (CTR) or an equivalent diet lacking serine and glycine (-SG) and treated with vehicle (Veh) or PH755 for 20 days. Data are presented as mean ± SEM (n=10 mice per group). (* $p < 0.05$, **** $p < 0.0001$; one-way ANOVA with Tukey's post hoc test).

(g). Representative images of Ki67-stained jejunum from C57BL/6J mice fed a control diet (CTR) or an equivalent diet lacking serine and glycine (-SG) and treated with vehicle (Veh) or PH755 for 20 days (n=5 mice per group).

Source data are provided as a Source Data file.

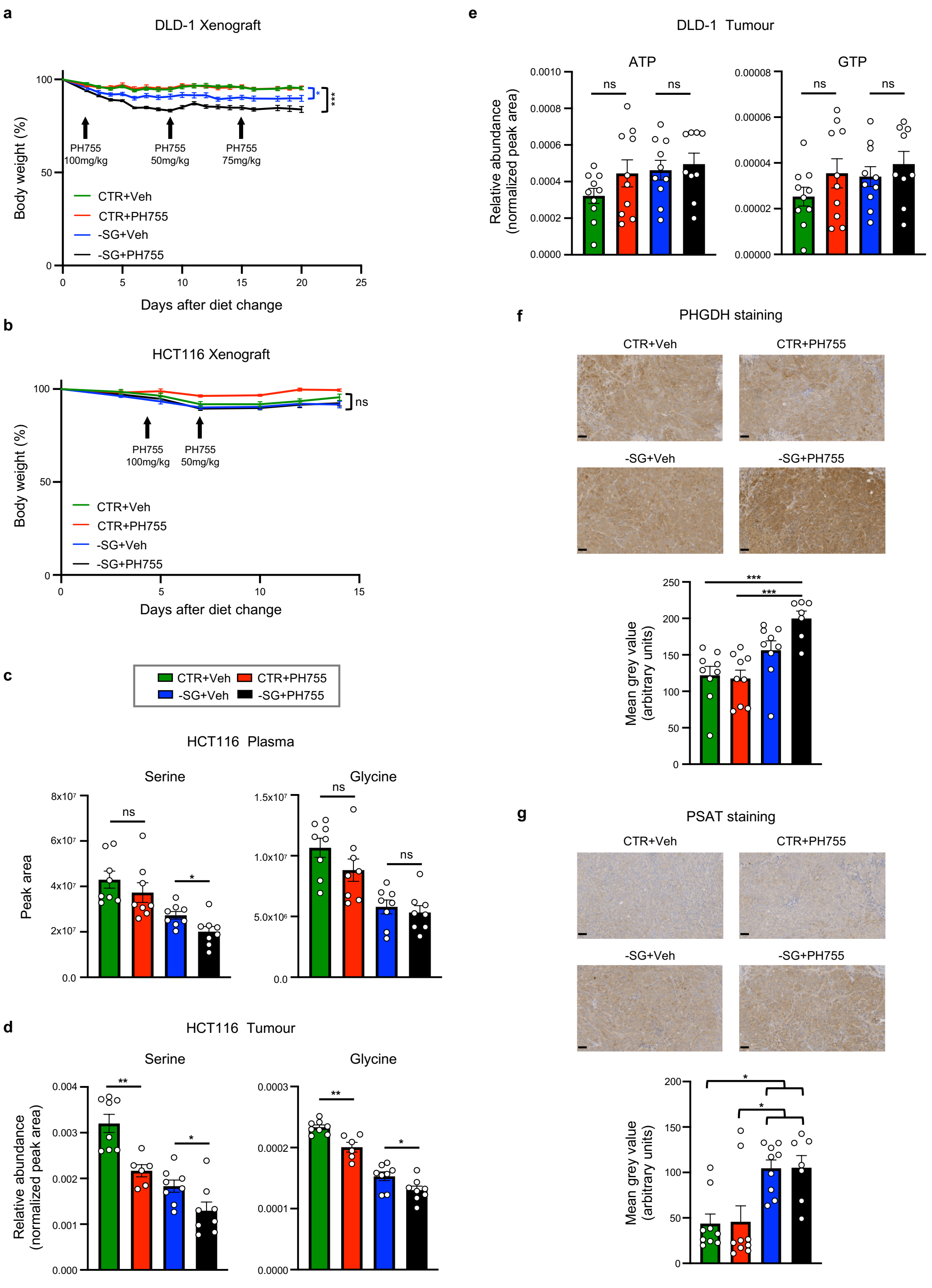

Supplementary Figure 7: ATF-4 response and metabolic alterations in tumours of mice treated with -SG diet and PH755.

(a&b). The weight of mice used in the DLD-1 (a) and HCT116 (b) xenograft experiments was recorded at regular intervals. Percentage of body weight was calculated from the initial weight taken the day of the diet change. Arrows show the starting day of the indicated treatment.

Data are presented as mean ± SEM. (ns: no significance, *$p < 0.05$; ***$p < 0.001$; two-way ANOVA plus Tukey's post hoc test). (a) CTR+Veh: n=10; CTR+PH755 n=10; -SG+Veh: n=10; -SG+PH755 n=9. (b) CTR+Veh: n=8; CTR+PH755 n=7; -SG+Veh: n=8; -SG+PH755 n=7 (n=number of mice).

(c). Plasma was taken at time of sacrifice from mice subcutaneously injected with HCT116 cells fed a control diet (CTR) or an equivalent diet lacking serine and glycine (-SG) and treated with vehicle (Veh) or PH755. LC-MS analysis was performed to evaluate serine and glycine content. Data are presented as mean ± SEM (n=8 mice per group). (* $p < 0.05$; unpaired two-sided Student *t* test).

(d). Serine and glycine levels measured by LC-MS in tumour lysates collected at end-point from animals subcutaneously injected with HCT116 cells. Peak area was normalized to total ion count (TIC). CTR+Veh: n=8; CTR+PH755 n=6; -SG+Veh: n=8; -SG+PH755 n=8 (n=number of mice). Data are presented as mean ± SEM. (* $p < 0.05$, ** $p < 0.01$; unpaired two-sided Student *t* test).

(e). ATP and GTP levels measured by LC-MS in tumour lysates collected at end-point from animals subcutaneously injected with DLD-1 cells. Peak area was normalized to total ion count (TIC). CTR+Veh: n=10; CTR+PH755 n=10; -SG+Veh: n=10; -SG+PH755 n=9 (n=number of mice). Data are presented as mean ± SEM. (ns: no significance; unpaired two-sided Student *t* test).

(f&g). Representative immunohistochemistry pictures and quantification of PHGDH staining (f) and PSAT staining (g) in DLD-1 tumours harvested at end-points from mice fed a control diet (CTR) or an equivalent diet lacking serine and glycine (-SG) and treated with vehicle (Veh) or PH755. CTR+Veh: n=9; CTR+PH755 n=9; -SG+Veh: n=9; -SG+PH755 n=7 (n=number of mice). Data are presented as mean ± SEM. (*$p < 0.05$, ***$p < 0.001$; one-way ANOVA with Tukey's post hoc test). Scale bar represents 50 μm.

Source data are provided as a Source Data file.
