# Supplementary material for: Serine synthesis pathway inhibition cooperates with dietary serine and glycine limitation for cancer therapy
Source: Nat Commun. 2021 Jan 14;12:366. doi: 10.1038/s41467-020-20223-y (PMC7809039; doi:10.1038/s41467-020-20223-y)
Supplement: Supplementary file 2 — Reporting summary [file 41467_2020_20223_MOESM2_ESM.pdf]

nature research

Corresponding author(s): Karen Vousden
Last updated by author(s): Oct 28, 2020

Reporting Summary

Nature Research wishes to improve the reproducibility of the work that we publish. This form provides structure for consistency and transparency in reporting. For further information on Nature Research policies, see our Editorial Policies and the Editorial Policy Checklist.

Statistics

For all statistical analyses, confirm that the following items are present in the figure legend, table legend, main text, or Methods section.

n/a	Confirmed	
☐	☒	The exact sample size (*n*) for each experimental group/condition, given as a discrete number and unit of measurement
☐	☒	A statement on whether measurements were taken from distinct samples or whether the same sample was measured repeatedly
☐	☒	The statistical test(s) used AND whether they are one- or two-sided<br>*Only common tests should be described solely by name; describe more complex techniques in the Methods section.*
☐	☒	A description of all covariates tested
☐	☒	A description of any assumptions or corrections, such as tests of normality and adjustment for multiple comparisons
☐	☒	A full description of the statistical parameters including central tendency (e.g. means) or other basic estimates (e.g. regression coefficient) AND variation (e.g. standard deviation) or associated estimates of uncertainty (e.g. confidence intervals)
☐	☒	For null hypothesis testing, the test statistic (e.g. *F*, *t*, *r*) with confidence intervals, effect sizes, degrees of freedom and *P* value noted<br>*Give P values as exact values whenever suitable.*
☒	☐	For Bayesian analysis, information on the choice of priors and Markov chain Monte Carlo settings
☒	☐	For hierarchical and complex designs, identification of the appropriate level for tests and full reporting of outcomes
☒	☐	Estimates of effect sizes (e.g. Cohen's *d*, Pearson's *r*), indicating how they were calculated

Our web collection on statistics for biologists contains articles on many of the points above.

Software and code

Policy information about availability of computer code

Data collection	LC-MS: Xcalibur 4.0.27.19; qPCR: QuantStudio™ 7 Flex Real-Time PCR System (software v1.3); Western blot: Image Studio Lite (version 5.2.5); Microscopy: Infinity Capture (version 6.5.4); IHC slides scanner: ZEISS ZEN 2.6 (blue edition)
Data analysis	Excel (version 16.16.26), GraphPad Prism 8 (version 8.3.1), ImageJ (version 2.0.0-rc-68/1.52e), LC-MS: TraceFinder 4.1 (version 4.1); Flow cytometry: FlowJo (version 10.5.2); IHC pictures: QuPath (version 0.1.2)

For manuscripts utilizing custom algorithms or software that are central to the research but not yet described in published literature, software must be made available to editors and reviewers. We strongly encourage code deposition in a community repository (e.g. GitHub). See the Nature Research guidelines for submitting code & software for further information.

Data

Policy information about availability of data

All manuscripts must include a data availability statement. This statement should provide the following information, where applicable:
- Accession codes, unique identifiers, or web links for publicly available datasets
- A list of figures that have associated raw data
- A description of any restrictions on data availability

The source data for the main and supplementary figures are provided as a Source Data file or can be found in the supplementary information file. All the other data supporting the findings of this study are available from the corresponding author upon reasonable request.

Field-specific reporting

Please select the one below that is the best fit for your research. If you are not sure, read the appropriate sections before making your selection.

- [x] Life sciences
- [ ] Behavioural & social sciences
- [ ] Ecological, evolutionary & environmental sciences

For a reference copy of the document with all sections, see nature.com/documents/nr-reporting-summary-flat.pdf

Life sciences study design

All studies must disclose on these points even when the disclosure is negative.

Sample size	Experiments were performed using sample sizes based on standard protocols in the field (see Diehl et al. Nat Metab. 2019 Sep;1(9):861-867, Luengo et al. Nat Commun. 2019 Dec 6;10(1):5604). No statistical test was performed to predetermine sample size. Mouse experiments all started with 10 or 8 (for HCT116 xenografts) animals per group. Occasionally a mouse had to be euthanized before the end of the experiment and was therefore not included in the final tumour growth curve.
Data exclusions	No data were excluded.
Replication	Information provided in Figure legends.
Randomization	All metabolic data are assigned a random order before being injected through the LC-MS column.<br>For mouse experiments with treatment, cages of up to 5 mice were randomly assigned a treatment.
Blinding	The identity of each mouse was blinded when measurements were collected and histology slides were blinded for analysis.<br>For in vitro experiments, the investigators were not blinded for group allocation as the same investigator both planned and performed the experiment.

Reporting for specific materials, systems and methods

We require information from authors about some types of materials, experimental systems and methods used in many studies. Here, indicate whether each material, system or method listed is relevant to your study. If you are not sure if a list item applies to your research, read the appropriate section before selecting a response.

Materials & experimental systems

n/a	Involved in the study	
[ ]	[x]	Antibodies
[ ]	[x]	Eukaryotic cell lines
[x]	[ ]	Palaeontology and archaeology
[ ]	[x]	Animals and other organisms
[x]	[ ]	Human research participants
[x]	[ ]	Clinical data
[x]	[ ]	Dual use research of concern

Methods

n/a	Involved in the study	
[x]	[ ]	ChIP-seq
[ ]	[x]	Flow cytometry
[x]	[ ]	MRI-based neuroimaging

Antibodies

Antibodies used

Western blot:

Primary antibodies

PHGDH (13428) from Cell Signaling Technology, Ref: 08/2019, Lot: 1
ATF-4 (D4B8) (11815) from Cell Signaling Technology, Ref: 03/2019, Lot: 4
Phospho-eIF2α (Ser51) (D9G8)(3398) from Cell Signaling Technology, Ref: 09/2019, Lot: 6
Phospho-p70S6 kinase (Thr389) (108D2) (9234) from Cell Signaling Technology, Ref: 09/2015, Lot: 11
p70S6 kinase (9202) from Cell Signaling Technology, Ref: 07/2020, Lot: 20
c-Myc (D84C12) (5605) from Cell Signaling Technology, Ref: 02/2019, Lot: 12
HIF-1alpha (D2U3T) (14179) from Cell Signaling Technology, Ref: 03/2020, Lot: 3
Caspase-3 (9662) from Cell Signaling Technology, Ref: 10/2016, Lot: 18
Cleaved Caspase-3 (Asp175) (9661) from Cell Signaling Technology, Ref: 01/2018, Lot: 45
beta-Actin (13E5) (4970) from Cell Signaling Technology, Ref: 03/2019, Lot: 15
GCN2 (F-7) (sc-374609) from Santa Cruz Biotechnology, lot: K0419
eIF2α (D-3) (sc-133132) from Santa Cruz Biotechnology, lot: C1119
p53 (DO-1) (sc-126) from Santa Cruz Biotechnology, lot: F2117

Vinculin (7F9) (sc-73614) from Santa Cruz Biotechnology, lot: E1719
PSAT (ab96136) from Abcam, lot: GR265773-22
PSPH (ab96414) from Abcam, lot: GR8276-10
Phospho-GCN2 (Thr899) (ab75836) from Abcam, lot: GR280045-22
ASNS (HPA029318) from Atlas Antibodies, lot: D105685
Puromycin (clone 12D10) (MABE343) from Sigma-Aldrich, lot: 3379285

Secondary antibodies

Anti-rabbit IgG, HRP-linked Antibody (7074) from Cell Signaling Technology, Ref: 12/2019, Lot: 28
Anti-mouse IgG, HRP-linked Antibody (7076) from Cell Signaling Technology, Ref: 04/2020, Lot: 35
IRDye 800CW Donkey anti-Rabbit IgG (926-32213) from LI-COR, lot: C90806-09
IRDye 800CW Donkey anti-Mouse IgG (926-32212) from LI-COR, lot: D00226-15
IRDye 680LT Donkey anti-Goat IgG (926-68024) from LI-COR, lot: C60614-05

IHC:
PHGDH (HPA021241) from Sigma-Aldrich, lot: 000000524
PSAT1 (PA5-22124) from Thermofisher, lot: UJ2858287B
Active Caspase-3 (AF835) from R&D Systems, lot: CFZ3519041
Ki67 SP6 (ab16667) from Abcam, lot: GR3313195-42

Flow cytometry:
APC anti-BrdU antibody (51-23619L) from BD Pharmingen, lot: 9213520

Validation

All antibodies described in this study were verified and confirmed for species reactivity and application as per the manufacturers' disclosure.

Western blot:

PHGDH (13428) from Cell Signaling Technology. Species reactivity: multiple species including human, validated for Western blot by manufacturer: https://www.cellsignal.com/products/primary-antibodies/phgdh-antibody/13428?Ntk=Products&Ntt=13428

ATF-4 (D4B8) (11815) from Cell Signaling Technology. Species reactivity: multiple species including human, validated for Western blot by manufacturer: https://www.cellsignal.com/products/primary-antibodies/atf-4-d4b8-rabbit-mab/11815?Ntk=Products&Ntt=11815

Phospho-eIF2α (Ser51) (D9G8)(3398) from Cell Signaling Technology. Species reactivity: multiple species including human, validated for Western blot by manufacturer: https://www.cellsignal.com/products/primary-antibodies/phospho-eif2a-ser51-d9g8-xp-rabbit-mab/3398?Ntk=Products&Ntt=3398

Phospho-p70S6 kinase (Thr389) (108D2) (9234) from Cell Signaling Technology. Species reactivity: multiple species including human, validated for Western blot by manufacturer: https://www.cellsignal.com/products/primary-antibodies/phospho-p70-s6-kinase-thr389-108d2-rabbit-mab/9234?Ntk=Products&Ntt=9234

p70S6 kinase (9202) from Cell Signaling Technology. Species reactivity: multiple species including human, validated for Western blot by manufacturer: https://www.cellsignal.com/products/primary-antibodies/p70-s6-kinase-antibody/9202?Ntk=Products&Ntt=9202

c-Myc (D84C12) (5605) from Cell Signaling Technology. Species reactivity: multiple species including human, validated for Western blot by manufacturer: https://www.cellsignal.com/products/primary-antibodies/c-myc-d84c12-rabbit-mab/5605?Ntk=Products&Ntt=5605

HIF-1alpha (D2U3T) (14179) from Cell Signaling Technology. Species reactivity: multiple species including human, validated for Western blot by manufacturer: https://www.cellsignal.com/products/primary-antibodies/hif-1a-d2u3t-rabbit-mab/14179?Ntk=Products&Ntt=14179

Caspase-3 (9662) from Cell Signaling Technology. Species reactivity: multiple species including human, validated for Western blot by manufacturer: https://www.cellsignal.com/products/primary-antibodies/caspase-3-antibody/9662?Ntk=Products&Ntt=9662

Cleaved Caspase-3 (Asp175) (9661) from Cell Signaling Technology. Species reactivity: multiple species including human, validated for Western blot by manufacturer: https://www.cellsignal.com/products/primary-antibodies/cleaved-caspase-3-asp175-antibody/9661?Ntk=Products&Ntt=9661

beta-Actin (13E5) (4970) from Cell Signaling Technology. Species reactivity: multiple species including human, validated for Western blot by manufacturer: https://www.cellsignal.com/products/primary-antibodies/b-actin-13e5-rabbit-mab/4970?Ntk=Products&Ntt=4970

GCN2 (F-7) (sc-374609) from Santa Cruz Biotechnology. Species reactivity: human, validated for Western blot by manufacturer: https://datasheets.scbt.com/sc-374609.pdf

eIF2α (D-3) (sc-133132) from Santa Cruz Biotechnology. Species reactivity: multiple species including human, validated for Western

blot by manufacturer: https://datasheets.scbt.com/sc-133132.pdf

p53 (DO-1) (sc-126) from Santa Cruz Biotechnology. Species reactivity: multiple species including human, validated for Western blot by manufacturer: https://datasheets.scbt.com/sc-126.pdf

Vinculin (7F9) (sc-73614) from Santa Cruz Biotechnology. Species reactivity: multiple species including human, validated for Western blot by manufacturer: https://datasheets.scbt.com/sc-73614.pdf

PSAT (ab96136) from Abcam. Species reactivity: human, validated for Western blot by manufacturer: https://www.abcam.com/phosphoserine-aminotransferase-antibody-ab96136.html

PSPH (ab96414) from Abcam. Species reactivity: human, validated for Western blot by manufacturer: https://www.abcam.com/psph-antibody-ab96414.html

Phospho-GCN2 (Thr899) (ab75836) from Abcam. Species reactivity: human, validated for Western blot by manufacturer: https://www.abcam.com/gcn2-phospho-t899-antibody-epr2320y-ab75836.html

ASNS (HPA029318) from Atlas Antibodies. Species reactivity: human, validated for Western blot by manufacturer: https://www.atlasantibodies.com/api/print_datasheet/HPA029318.pdf

Puromycin (clone 12D10) (MABE343) from Sigma-Aldrich. Species reactivity: human, validated for Western blot by manufacturer: https://www.sigmaaldrich.com/catalog/product/mm/mabe343?lang=en&region=GB

IHC:

PHGDH (HPA021241) from Sigma-Aldrich. Species reactivity: multiple species including human, validated for immunohistochemistry by manufacturer: https://www.sigmaaldrich.com/catalog/product/sigma/hpa021241?lang=en&region=GB

PSAT1 (PA5-22124) from Thermofisher. Species reactivity: multiple species including human, validated for immunohistochemistry by manufacturer: https://www.thermofisher.com/order/genome-database/dataSheetPdf?producttype=antibody&productsubtype=antibody_primary&productId=PA5-22124&version=123

Active Caspase-3 (AF835) from R&D Systems. Species reactivity: multiple species including human, validated for immunohistochemistry by manufacturer: https://resources.rndsystems.com/pdfs/datasheets/af835.pdf

Ki67 SP6 (ab16667) from Abcam. Species reactivity: multiple species including human, validated for immunohistochemistry by manufacturer: https://www.abcam.com/ki67-antibody-sp6-ab16667.html

Eukaryotic cell lines

Policy information about cell lines

Cell line source(s)	All cell lines (HT-29, SW48, SW480, SW620, CACO2, HCT116, RKO, VACO5, MDA-MB-468, DLD-1, HCT-15, SW1417, LoVo and CL-34 cells) were provided by the Cell Services repository for the Francis Crick Institute.
Authentication	All cell lines were authenticated using STR profiling and species identifications.
Mycoplasma contamination	All cell lines were negative for mycoplasma upon thawing.
Commonly misidentified lines (See ICLAC register)	No commonly misidentified cell lines were used in the study.

Animals and other organisms

Policy information about studies involving animals; ARRIVE guidelines recommended for reporting animal research

Laboratory animals	- C57BL/6J male mice (obtained from Charles River, 14 weeks old)<br>- CD-1 female nude mice (obtained from Charles River, 7-9 weeks old)
Wild animals	This study did not involve wild animals.
Field-collected samples	This study did not involve samples collected from the field.
Ethics oversight	All experiments were conducted in compliance with the UK Home Office-approved project licences and personal licences (Animals Scientific Procedures Act 1986) and within institutional welfare guidelines (Francis Crick Institute and CRUK Beatson Institute).

Note that full information on the approval of the study protocol must also be provided in the manuscript.

Flow Cytometry

Plots

Confirm that:

- [x] The axis labels state the marker and fluorochrome used (e.g. CD4-FITC).
- [x] The axis scales are clearly visible. Include numbers along axes only for bottom left plot of group (a 'group' is an analysis of identical markers).
- [x] All plots are contour plots with outliers or pseudocolor plots.
- [x] A numerical value for number of cells or percentage (with statistics) is provided.

Methodology

Sample preparation	HCT116 and DLD-1 cells were grown for 48 hours in -SG medium or CM and treated with 10µM PH755 diluted in DMSO or DMSO alone. To determine the percentage of bromodeoxyuridine (BrdU) positive cells, 10µM BrdU was then added to culture media for an additional 5 hours while for cell cycle analysis, 10µM BrdU was added for only 30 minutes. Cells were then harvested, fixed and stained with APC anti-BrdU antibody (and 7-AAD for cell cycle analysis) using the APC BrdU Flow kit (BD Pharminogen, Cat no: 552598) following the manufacturer's instructions.
Instrument	Fortessa flow Cytometer
Software	The analysis was performed using FlowJo 10.5.2.
Cell population abundance	Cells were not sorted.
Gating strategy	For graphs representing the percentage of BrdU positive cells, cells were first detected in SSC-A/FSC-A. Following this, doublets were gated out by plotting SSC-A/SSC-W. BrdU positive cells were then assessed in FSC-A/BrdU-APC. The control media condition was used to determine the BrdU negative and BrdU positive cells.<br>For graphs representing cell cycle analysis, cells were first detected in SSC-A/FSC-A. Following this, doublets were gated out by plotting SSC-A/SSC-W. Cells were then plotted for Brdu-APC/7-AAD. G1 and subG1 were gated on Brdu-/7AAD low and Brdu-/7-AAD- cells, G2 was gated on Brdu-/7AAD high population, S was gated on BrdU+ cells.

- [x] Tick this box to confirm that a figure exemplifying the gating strategy is provided in the Supplementary Information.
